# Breast tumor IGF1R regulates cell adhesion and metastasis: alignment of mouse single cell and human breast cancer transcriptomics

**DOI:** 10.3389/fonc.2022.990398

**Published:** 2022-12-07

**Authors:** Alison E. Obr, Joseph J. Bulatowicz, Yun-Juan Chang, Virginia Ciliento, Alexander Lemenze, Krystopher Maingrette, Quan Shang, Emily J. Gallagher, Derek LeRoith, Teresa L. Wood

**Affiliations:** ^1^ Department of Pharmacology, Physiology & Neuroscience, New Jersey Medical School, Rutgers University, Newark, NJ, United States; ^2^ Office of Advance Research Computing, Rutgers University, Piscataway, NJ, United States; ^3^ Department of Pathology, New Jersey Medical School, Rutgers University, Newark, NJ, United States; ^4^ Division of Endocrinology, Diabetes and Bone Diseases, The Samuel Bronfman Department of Medicine, Icahn Sinai School of Medicine at Mt. Sinai, New York, NY, United States

**Keywords:** insulin-like growth factor receptor, metastasis, breast cancer, adhesion, cadherin

## Abstract

**Introduction:**

The acquisition of a metastatic phenotype is the critical event that determines patient survival from breast cancer. Several receptor tyrosine kinases have functions both in promoting and inhibiting metastasis in breast tumors. Although the insulin-like growth factor 1 receptor (IGF1R) has been considered a target for inhibition in breast cancer, low levels of IGF1R expression are associated with worse overall patient survival.

**Methods:**

To determine how reduced IGF1R impacts tumor phenotype in human breast cancers, we used weighted gene co-expression network analysis (WGCNA) of Molecular Taxonomy of Breast Cancer International Consortium (METABRIC) patient data to identify gene modules associated with low IGF1R expression. We then compared these modules to single cell gene expression analyses and phenotypes of mouse mammary tumors with reduced IGF1R signaling or expression in a tumor model of triple negative breast cancer.

**Results:**

WGCNA from METABRIC data revealed gene modules specific to cell cycle, adhesion, and immune cell signaling that were inversely correlated with IGF1R expression in human breast cancers. Integration of human patient data with single cell sequencing data from mouse tumors revealed similar pathways necessary for promoting metastasis in basal-like mammary tumors with reduced signaling or expression of IGF1R. Functional analyses revealed the basis for the enhanced metastatic phenotype including alterations in E- and P-cadherins.

**Discussion:**

Human breast and mouse mammary tumors with reduced IGF1R are associated with upregulation of several pathways necessary for promoting metastasis supporting the conclusion that IGF1R normally helps maintain a metastasis suppressive tumor microenvironment. We further found that reduced IGF1R signaling in tumor epithelial cells dysregulates cadherin expression resulting in reduced cell adhesion.

## Introduction

Metastatic breast cancer is the leading cause of death from breast cancer (1, 2). Several individual genes and associated cellular pathways contribute to a metastatic phenotype but the mechanisms that lead to metastasis are still poorly understood. Receptor tyrosine kinases (RTKs) have been implicated in promoting metastatic properties in tumor cells. RTK domain mutations are not a prominent feature in most cancers; instead, RTK expression level is the general driver of tumorigenesis and metastasis (3–6). A well-known RTK, HER2, has a prominent role in a subclass of breast cancers and has been the focus for successful cancer therapeutics. However, targeting several other RTKs including the epidermal growth factor receptor (EGFR) and the insulin-like growth factor receptor (IGF1R) in breast tumors has been mostly unsuccessful (4, 7, 8). The emerging theme for these receptors is their context- and/or cell-type-dependent functions that change whether they are growth-promoting or growth-inhibiting in the primary tumor or metastatic environment. For example, EGFR signaling promotes growth of primary mammary tumors but suppresses growth of lung metastatic tumors [for review, see (4)]. In the case of the IGF1R, results from mouse models also support a dual function in primary tumor formation and metastasis suppression which may be due to differential actions on proliferation or differentiation depending on the tumor lineage [for review, see (9)].

Expression of IGF1R has been implicated in tumor oncogenesis by promoting tumor cell proliferation and survival (10–12). Due to this oncogenic function, several IGF1R inhibitors have been developed and used in clinical trials. While IGF1R was a clear target, the inhibitors were largely unsuccessful in the clinic (7, 8). There is now evidence that the IGF1R also has tumor or metastasis suppressive functions; IGF1R expression in breast tumors correlates with positive overall patient survival and a more differentiated tumor phenotype (13–15). Consistent with these data, recent analyses using two different patient databases, The Cancer Genome Atlas (TCGA) and the Molecular Taxonomy of Breast Cancer International Consortium (METABRIC), have revealed low IGF1R expression is associated with undifferentiated, triple-negative breast cancer (TNBC) and worse overall survival (16, 17).

In the present study, we utilized the METABRIC patient database (18) and single-cell RNA sequencing of two IGF1R loss-of-function mouse tumor models to uncover how IGF1R signaling regulates intrinsic epithelial cell signaling to suppress metastasis. We identify key pathways necessary for promoting metastasis including downregulation of immune cell infiltration and function and altered tumor cell phenotype and adherence. Here, we show that IGF1R is required to maintain a metastasis suppressive tumor microenvironment. We further show that reduced IGF1R signaling in tumor epithelial cells dysregulates E- and P-cadherin resulting in reduced cell adhesion.

## Materials & methods

### Animal models

All animal protocols were approved by the Rutgers University Institutional Animal Care and Use Committee (Newark, NJ) and all experiments were managed in accordance with the NIH guidelines for the care and use of laboratory animals. Animal care was provided by the veterinary staff of the division of animal resources in the New Jersey Medical School Cancer Center of Rutgers Biomedical Health Sciences. The *MMTV-Wnt1* line on an FVB background [FVB.Cg-Tg(Wnt1)1Hev/J] was obtained as a gift from Dr. Yi Li. The *MMTV-Wnt1//MMTV-dnIgf1r* (referred to here as DN-Wnt1) line was described previously (19).

Mice carrying floxed alleles of exon 3 of the *Igf1r* gene (20) were bred with a keratin 8 (K8)-Cre^ERT^ transgenic line (JAX stock #017947) (21) and with the *MMTV-Wnt1* transgenic line to produce female mice that were homozygous for the *Igf1r* floxed alleles and hemizygous for both the K8-Cre^ERT^ and *MMTV-Wnt1* transgenes referred to as K8iKOR-Wnt1 mice.

### K8iKOR-Wnt1 tamoxifen dosage paradigm

The tamoxifen dosage paradigm was determined following a developmental study of the effect of tamoxifen on mammary gland development. Three doses of tamoxifen, 5 mg, 2 mg, 1.5 mg or sesame oil were administered once per day for 3 consecutive days in 4-week-old or 8-week-old FVB mice. Four weeks post-injection, mammary gland development was observed using Carnoy’s fixative to clear whole mounted mammary glands. Mammary glands from control samples injected with sesame oil demonstrated no significant changes in secondary or tertiary branching compared to naïve glands, while mammary gland development was stunted with the 5 mg dose of tamoxifen administered at 4 weeks of age. Similar to 4 weeks of age, mammary gland branching was stunted at 8 weeks of age with the 5 mg dose of tamoxifen but not with lower tamoxifen doses. Thus, for all tumor studies, tamoxifen (2 mg for 3 consecutive days) was administered at the end of puberty (8 weeks) to avoid disturbing mammary gland development (22, 23) and as confirmed in our studies. Age-matched (8 weeks) females were injected with vehicle sesame oil (control) or tamoxifen for 3 consecutive days to delete the floxed *Igf1r* alleles. Controls for tumor studies included K8-Cre^ERT^ positive females injected with vehicle or K8-Cre^ERT^ negative females injected with tamoxifen. No differences were detected between vehicle and tamoxifen injected controls thus these were combined unless otherwise noted in the methods. Lungs and tumors were harvested when they reached 1.5 cm^3^. We confirmed deletion of *Igf1r* K8iKOR-Wnt1 by qRT-PCR for *Igf1r* expression (**Supp. Figure 2**) and expression of the exon 4 deletion-specific *Igf1r* transcript in tumors and in FAC-sorted luminal epithelial cells.

### Tumor latency and growth curves

Wnt1 and K8iKOR-Wnt1 female mice were palpated every five days for tumors beginning at nine weeks of age or 1 wpi sesame oil or tamoxifen. Since no differences in latency were observed between vehicle and tamoxifen injected controls, we combined these animals for these studies. Tumor growth was measured by caliper bi-weekly once a tumor was identified, and the mouse was sacrificed when the tumor reached 1.5 cm^3^.

### Mammary tumor epithelial cell dissociation

Tumor mammary epithelial cells (MECs) were isolated from Wnt1, DN-Wnt1, and K8iKOR-Wnt1 mice similarly to our prior study (19). Whole tumors were excised and dissociated with the gentleMACs tissue dissociator (130-093-235, protocol m_TDK2) and mouse specific tumor dissociation kit (Miltenyi, 130-096-730). Organoids that retained basement membrane attachments were trypsinized (0.05% Trypsin-EDTA, Gibco) and filtered with a 40 mm cell strainer (BD Biosciences) to isolate a single cell suspension of dissociated tumor MECs. Isolated tumor MECs were counted with a hemocytometer for flow cytometry, FACS, *in vitro* adhesion assays, and cell culture assays.

### Sorting of mammary tumor epithelial cells

Tumor MECs from either Wnt1 or DN-Wnt1 mice (n=4) were isolated for single cells as described above with minor adjustments for depletion of unnecessary cells. Red blood cells were lysed with a lysis buffer (155 mM NH_4_Cl, 12 mM NaHCO_3_, 0.1 mM EDTA) for 5 minutes. Tumor MECs were resuspended at 10^6^ cells/ml in FACS buffer (2% BSA, 2% goat serum in PBS) and immunolabeled with fluorochrome-conjugated cell surface antibodies as described in our previous studies (19). Single cells were prepared for FACS as previously described (24) and sorted at 70 psi using a 70-um nozzle on the Beckton Dickenson FACS Aria directly into PBS.

### Flow cytometry analysis of lineage-specific tumor epithelial cells

Tumor MECs from K8iKOR-Wnt1 mice injected with sesame oil or tamoxifen were isolated for single cells as described above. Since no differences in flow cytometry analysis were observed between vehicle and tamoxifen injected controls, we combined these animals. Tumor MECs were immunolabled with fluorochrome-conjugated cell surface antibodies at 1x10^6^ cells/100ul FACS buffer as described in our previous studies (17, 19). Cells were labelled for viability using a Live/Dead dye (Invitrogen, L34958) and fixed with 1% paraformaldehyde. Single cells were analyzed using the BD LSRFortessa flow cytometer.

### RNA isolation and real-time quantitative PCR

RNA was purified from whole tumor and sorted tumor epithelial cells according to the manufacturer’s protocol (Qiagen). RNA concentration and quality was assayed with the NanoDrop ND-1000 (Thermo Scientific). Epithelial cell and sorted tumor epithelial cell cDNA was transcribed according to manufacturer’s protocol using SuperScript II (Invitrogen) from total RNA (200 ng). Samples were run in technical triplicate to determine relative gene expression by real-time quantitative PCR (qRT-PCR) detected with SsoAdvanced Universal SYBR Green Supermix (BioRad) using the BioRad CFX96 real-time PCR machine according to manufacturer’s instructions. Transcript levels were normalized to glyceraldehyde-3-phosphate dehydrogenase (GAPDH) or Gusb for mouse and ß-actin for human, and data were analyzed using the Q-Gene software (BioTechniques Software Library) (25). Primer oligonucleotide pairs for qRT-PCR are provided (**Supp. Table 1**).

### Histology and immunofluorescence

Tumor tissues and lungs from animals with primary tumors (n=4 per genotype) were drop-fixed in 4% paraformaldehyde (PFA), embedded in paraffin, and sectioned at 7 µm. Lung sections from animals with primary tumors were used for hematoxylin and eosin staining. Tumor sections were processed for antigen retrieval for immunofluorescence (IF) as described previously (26). Tissue sections were immunostained with primary antibodies: E-cadherin (1:100; Invitrogen, ECCD-2), P-cadherin (1:100; Invitrogen, MA1-2003), and with species-specific fluorochrome-conjugated secondary antibodies (1:500, Invitrogen).

Fluorescent images were captured using an All-in-One Fluorescent Microscope BZ-X (Keyence, America), and BZ- scientific imaging processing software was used to capture images. At least 5 individual fields were captured at 20X or 40X magnification from tumor sections (n=3 per genotype; 3 sections per genotype averaged). For thicker sections, the Z-stack function was used to capture multiple images on the Z-axis. The Full-focus function was used to select areas at the sharpest focus and obtain the deconvoluted image.

### Counting macro and micrometastases in lung sections

Lung tissue from primary and TVI animals were sectioned at 7 µm through the entire lung. For coverage of the entire lung, 3 sections were taken and placed on slides and the next 3 sections were disposed through the entirety of the lung tissue or until reaching 72 individual sections. Representative sections (middle section of each 3 sections) were used for H&E staining. Individual macrometastases were counted by eye and micrometastases were counted at 10X magnification with a brightfield microscope (Olympus Provis AX70) from each H&E-stained slide (n=24).

### RNAscope analysis of dominant negative IGF1R expression

RNAScope Multiplex Fluorescent Assay v2 and a human IGF1R probe (Advanced Cell Diagnostics, Inc) was used to determine *dnIGF1R* RNA expression. Tumor tissues were fixed in 4% PFA, paraffin embedded, and sectioned at 7 µm. Tissue samples were deparaffinized and pretreated with hydrogen peroxide, antigen retrieval, and protease plus reagents. (Mild Reagents Timepoint; RNAScope). Tissue sections were incubated at 40°C (Isotemp Incubator, Fisher Scientific) with either Hs-IGF1R-No-XMm probe (Cat No. 471961), Negative probe (Cat No. 320871), or Positive Probe (Cat No 320881). The probe signal was amplified using Amplification Reagents (RNAScope) and signal was developed using the Multiplex FL v2 HRP-C1, HRP blocker, and Opal 620 fluorophore (Akoya Biosciences, FP1495001KT, 1:3000). Sections were incubated with DAPI (RNAScope) and mounted with ProLong Gold Antifade Mounting medium (Invitrogen). Images were captured on the Keyence BZ-X at 40x and 60x magnification.

### Tumor epithelial cell *in vitro* adhesion assays

Primary tumors were dissociated as described above and incubated in tissue culture on collagen coated plates for 10 hours. Culture media (DMEM/F12, 5% FBS, insulin (5 μg/mL), EGF (5 ng/mL), hydrocortisone (1 μg/mL), 0.1% gentimicin) was removed and cells in suspension were fixed on slides using a cytospin (Shandon Cytospin 3) for 10 minutes at 1500 rpm for immunofluorescence (IF). Cells attached to the collagen matrix were fixed with 4% PFA for 10 minutes at room temperature for IF analysis or lysed with RLT buffer (Qiagen) for RNA isolation and qRT-PCR analysis as described above.

For IF, cells were processed for staining as previously described (27). Cells were stained with primary antibodies: cytokeratin-8 (1:100; TROMA-I, DSHB) and cytokeratin-14 (1:250; Invitrogen, PA5-16722) and with species-specific fluorochrome-conjugated secondary antibodies (1:500, Invitrogen). To visualize cell nuclei, cells were stained with DAPI (1:10,000 in PBS). Images were captured as described above and cells were manually counted using ImageJ.

### Single-cell RNA sequencing

Whole Wnt1 (tamoxifen injected, Cre negative), DN-Wnt1, and K8iKOR-Wnt1 tumors were dissociated as described above and tumor cells were filtered with a 70 μm filter directly after dissociation to collect single cells from the entire tumor. Cells were captured using the 10X Chromium system (10X Genomics) and sequenced with the NextSeq 500 (Illumina). Raw reads were barcode deconvoluted and aligned to the reference genome (mm10) *via* cellranger (v3.1.0). All subsequent processing was performed using the Seurat package within R (v3.1.5). Low quality cells (cells with percentage of reads of mitochondrial origin >10%, with percentage of reads of ribosomal origin >45%, with <1000 feature counts, with >6000 feature counts) were filtered from the dataset, and read counts were normalized using the scTransform method (28). Samples were integrated with the Seurat integrate function (29) and clustered *via* UMAP according to nearest neighbors. Re-clustering was performed as above on subset clusters based on common annotation types.

### WGCNA analysis of METABRIC data for gene module identification

The data generated from 1981 patients within the METABRIC project (18) was used in this investigation. These data were accessed through Synapse (synapse.sagebase.org), including normalized expression data and clinical feature measurements. The associated expression Z scores were downloaded from cBioPortal (30, 31) (https://www.cbioportal.org/). The method of weighted gene co-expression network analysis (WGCNA) (32, 33) was used to identify gene modules with significant statistical association to the phenotypic trait including patient age, tumor size, tumor grade, cancer subtype, and IGF1R expression as Z score.

The analysis was performed within R environment, version 3.6.0, and WGCNA v. 1.68. First, genes with higher expression variance among patient samples (above its quantile) were filtered, resulting in a total of 12394 out of 49576 genes selected. Then, a gene co-expression network was constructed with expression values (normalized) of the selected genes, followed by an adjacency matrix to describe the correlation strength between the nodes. Subsequently, the adjacency matrix was transformed into a topological overlap matrix (TOM), which is a method to quantitatively describe the similarity in nodes by comparing the weighted correlation between two nodes and other nodes. The hierarchical clustering was then applied to identify modules, each containing at least 30 genes (minModuleSize = 30). Finally the eigengene was calculated, the modules were hierarchically clustered, and similar modules were merged (mergeCutHeight = 0.25). A soft-threshold of 6 was chosen which was the lowest power that resulted in a scale free topology fit index to be above 0.9. The correlation between the modules and the clinical data was calculated to identify significant modules correlated with the clinical trait.

### Ingenuity pathway analysis

scRNA-seq: Differentially expressed gene sets were identified from the DN-Wnt1 and K8iKOR-Wnt1 compared to Wnt1 mouse tumors for each whole tumor and epithelial cell specific cluster determined from scRNA-seq as described above. These differentially expressed genes were used for IPA enrichment and graphical summary analysis. The top 5 pathways based on significance were plotted by percent genes altered in each pathway. Graphical summaries were generated using the top pathways, cell functions, and target genes identified from differentially expressed genes (DN-Wnt1 vs. Wnt1; K8iKOR-Wnt1 vs. Wnt1) in each cluster.

WGCNA METABRIC analysis: Gene names and expression levels identified from highly correlative co-expression gene modules identified in the WGCNA analysis were uploaded into the IPA software (Qiagen) and analyzed for pathway enrichment. The top 5 pathways based on log-fold change significance for each module were plotted in GraphPad by percentage of total genes up- and down-regulated in each pathway.

Comparison Analysis: Whole tumor gene changes were compared to ME genes where the output is pathway alterations. Here, exact genes were not completely similar, but pathways were comparable.

### Statistics

All graphical data were expressed as the mean ± SEM. Statistical comparisons were carried out by GraphPad Prism9 software. The Student’s *t*-test or non-parametric Mann-Whitney U test was used for two-group comparisons. Specific comparisons are described in figure legends when necessary. For multiple variable analysis, the One-Way ANOVA with Tukey’s Multiple Comparison *post-hoc* test was performed. For the tumor growth curve and *in vitro* adhesion analysis, the non-linear regression least squares regression for slope best fit was used to compare differences between each line. The Chi-Square test was used to determine differences between genotypes in the metastasis table. Power calculations were performed based on pilot data to determine the number of tumor samples necessary using a 2-sided hypothesis test, an a = 0.0025, and 80% power.

## Results

### Low levels of IGF1R correlate with a metastatic gene signature in breast cancer

Recent analysis of TCGA and METABRIC databases have revealed IGF1R expression is reduced in TNBC (16, 17). Furthermore, low levels of IGF1R predict worse overall patient survival across all breast cancer subtypes (17, 34). Recently, we used the human METABRIC database to stratify low and high IGF1R expressing tumors with lymph node positivity, a readout of early-stage metastasis. These analyses revealed that lymph node positivity is ~20% higher in human breast tumors with low IGF1R expression versus those with high IGF1R expression (9). Our previous studies reported IGF1R expression levels in human tumors are inversely correlated with several key target genes that alter the tumor microenvironment (17). These expression analyses of human breast tumors with low IGF1R were performed with genes we identified as dysregulated in our mouse tumor model with reduced IGF1R signaling (17, 19). The findings from human and mouse support the hypothesis that low expression of IGF1R could be used to identify gene signatures associated with aggressive breast cancers. Network-based systems biology has become an important method for analyzing high-throughput gene expression data and gene function mining. One of the well-recognized methods, weighted gene co-expression network analysis (WGCNA), generates not only gene co-expression networks, but also a derived partitioning of clusters of genes (modules) and identifies the central players within the modules (32, 33). To independently stratify genes correlated with either low or high IGF1R expression in human breast cancers, we performed a global unbiased WGCNA utilizing the METABRIC database to identify gene expression modules associated with IGF1R expression Z-score, referred to as IGF1R gene set 1 (IGF1R-GS1; **Supp. Figure 1A**). The modules with the highest correlation were then used to identify relevant pathways using ingenuity pathway analysis (IPA) (IGF1R-GS1; **Supp. Figures 1B–H**).

**Figure 1:**
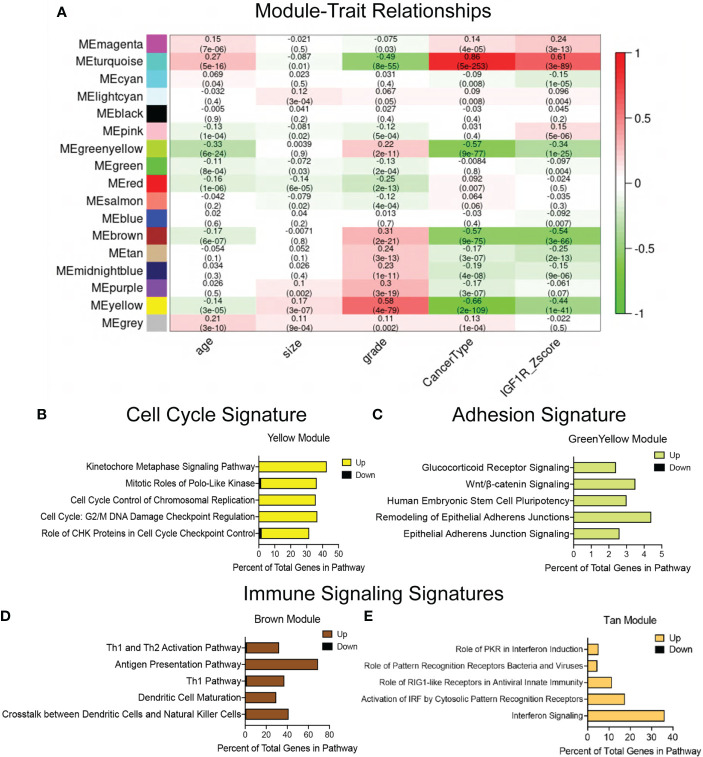
Defining gene signatures associated with IGF1R expression and tumor phenotype in human breast cancers. **(A)** Table of refined integrated WGCNA (IGF1R-GS2) showing module and clinical trait association. Each row corresponds to a module eigengene (ME), each column to a clinical measurement. Each cell contains the corresponding correlation and p-value (in parentheses). The table is color-coded by correlation according to the color legend. Green < 0 for negative correlation; Red > 0, for positive correlation. **(B–E)** Top 5 pathways identified by ingenuity pathway analysis (IPA) revealing key signatures in 4 modules inversely correlated with IGF1R expression. (yellow module=cell cycle signature, greenyellow module=adhesion signature, brown and tan modules=immune signaling signatures).

Due to the large number of genes and pathways altered in the IGF1R-GS1, we refined our WGCNA analyses to limit the original data set to those genes with the strongest positive or negative correlation to IGF1R expression (**Figure 1A**). In this refined gene set (IGF1R-GS2), we identified four gene co-expression modules significantly correlated with low IGF1R (correlation score ≤ -0.25), all of which were also associated with high tumor grade and three of which were associated with TNBC. One additional module significantly associated with high IGF1R (correlation 0.61) was also associated with ER+/PR+ breast cancers and low tumor grade (**Figure 1A**).

We then used IPA on the genes from individual modules identified in IGF1R-GS2 to define the pathways associated with the lowest IGF1R Z-scores. These analyses revealed genes involved in control of cell cycle checkpoint regulation and chromosome replication (yellow, Cell Cycle Signature; **Figure 1B**), and in epithelial adherens junctions (green-yellow, Adhesion Signature; **Figure 1C**). The two additional modules associated with low IGF1R contained genes involved in immune cell signaling (brown, tan; **Figures 1D, E**). Taken together, these findings indicate that reduced IGF1R in breast tumors is associated with alterations in intrinsic tumor epithelial cell pathways as well as extrinsic immune microenvironment signatures that promote metastasis.

A major question that arises from the METABRIC WGCNA is whether there is a causative relationship between IGF1R expression and associated gene alterations and, ultimately, phenotype of breast cancer. We published previously that low IGF1R expression predicts poor patient survival across all breast cancer subtypes (17, 19) suggesting negative functional consequences from loss of IGF1R expression. Our goal in this study was to use mouse models to test the hypothesis from the human data that low IGF1R in breast tumors directly contributes to a metastatic phenotype through dysregulated expression of specific cellular pathways.

### Mammary epithelial cell specific IGF1R deletion promotes Wnt1 driven tumor metastasis

**Figure 2:**
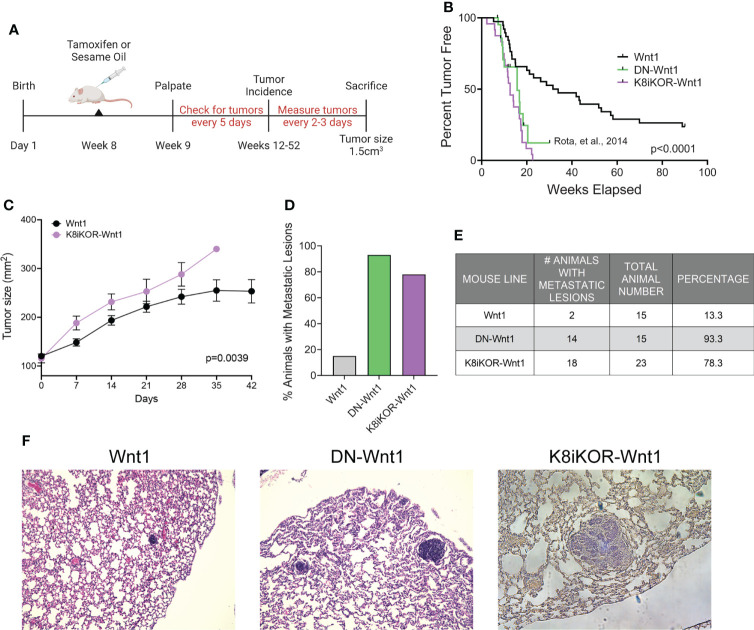
Luminal loss of IGF1R decreases tumor latency and increases metastasis. **(A)** Schematic for luminal lineage *Igf1r* knockout. **(B)** Latency curve for tumor development in Wnt1, DN-Wnt1, and K8iKOR-Wnt1 animals. For K8iKOR-Wnt1 animals, tumor latency is weeks post tamoxifen injection. *Statistic:* Mann-Whitney test **(C)** Growth curve after tumors arise until time of euthanization. *Statistic:* Non-linear regression best fit for line slopes. **(D, E)** Graph of the percentage of animals **(D)** and table of number of animals **(E)** with metastatic lesions after establishment of a primary tumor. *Table Statistic*: Chi-square test; p = 0.0251 for Wnt1 vs. DN-Wnt1 and K8iKOR-Wnt1. For Wnt1 controls, vehicle and tamoxifen injected animals were combined as the phenotypes were equivalent. **(F)** Micrograph images showing examples of metastases in H&E stained lung sections from Wnt1, DN-Wnt1 and K8iKOR-Wnt1 mice with primary tumors.

To test how loss of IGF1R alters the primary tumor phenotype, we made use of two distinct mouse models. In one model developed previously in our lab, IGF1R function is reduced through mammary epithelial expression of a dominant-negative human *IGF1R* transgene (MMTV-*dn*IGF1R) in the *MMTV-Wnt1* (Wnt1) basal-like breast cancer tumor model [DN-Wnt1; (19);]. In this mouse line, the loss of IGF1R function results in decreased tumor latency and increased lung metastases, while tumor growth is unchanged (19). To model human breast cancers with low IGF1R expression, we also generated a mammary luminal epithelial lineage-specific *Igf1r* knockout mouse driven from a tamoxifen-inducible Keratin 8 (K8)-Cre, referred to as the K8iKOR line (**Figure 2A**). Loss of *Igf1r* was verified in mammary epithelial cells (MECs) isolated from hyperplastic glands in 16-week-old virgin K8iKOR-Wnt1 mice compared to control, Wnt1 mice (**Supp. Figure 2A**). Decreased *Igf1r* gene expression was maintained in tumors of the K8iKOR-Wnt1 line (**Supp. Figure 2B**).

To determine the effects of luminal epithelial specific *Igf1r* gene deletion in Wnt1-driven mammary tumorigenesis, we assessed tumor latency rates in the K8iKOR-Wnt1 mouse line compared to the control Wnt1 line and to our prior tumor latency data on the DN-Wnt1 mouse line (19). The mean tumor latency of Wnt1 mice was consistent with previous reports (35, 36), where 50% of control Wnt1 animals formed palpable tumors at 41.7 weeks of age (**Figure 2B**). Tumor latency was significantly decreased in K8iKOR-Wnt1 mice (12.5 weeks after tamoxifen injection, p<0.0001) (**Figure 2B**) similar to the DN-Wnt1 mouse line as previously reported (16.6 weeks, p<0.0001) (**Figure 2B**) (19). Once tumors formed, tumor growth was significantly increased in K8iKOR-Wnt1 compared to control Wnt1 tumors (**Figure 2C**). These data indicate that decreased expression of *Igf1r* in luminal epithelial cells accelerates tumor initiation as well as tumor growth in the context of elevated Wnt signaling.

Although the Wnt1 tumors model a basal-like TNBC, these tumors have low metastatic potential (35). In contrast, loss of luminal epithelial *Igf1r* in the Wnt1 tumors significantly increased the percentage of animals with lung micrometastases (from 13.3% to 78.3%) similar to the high metastatic rate (93.3%) in the DN-Wnt1 mice (**Figures 2D–F**). Thus, either reduced *Igf1r* expression or reduced IGF1R function in mammary epithelium promotes metastasis of the primary Wnt1 tumor cells.

### Single-cell sequencing of mammary tumors to analyze epithelial IGF1R function in regulating tumor cell heterogeneity

**Figure 3:**
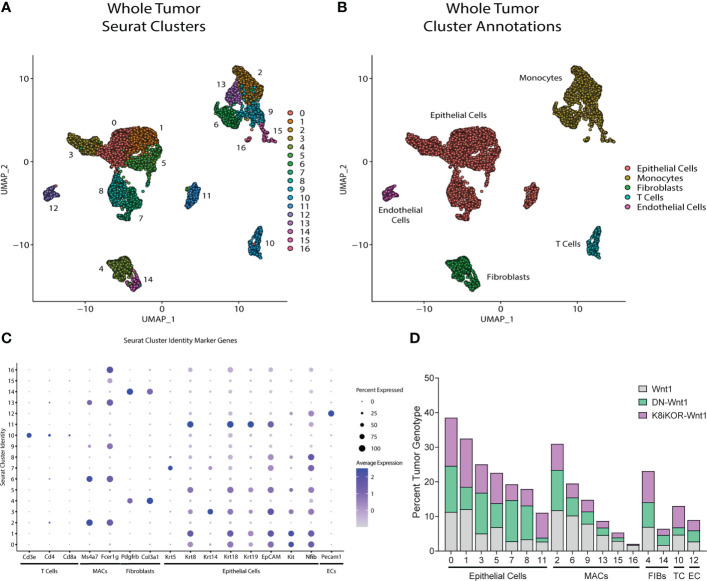
Identifying mammary tumor heterogeneity by single cell RNA-sequencing. **(A)** Uniform Manifold Approximation and Projection (UMAP) plot of cells from Wnt1, DN-Wnt1, and K8iKOR-Wnt1 tumors resulting in 17 individual clusters. **(B)** UMAP plot with identification of cluster cell types defined by known markers. **(C)** Dot plot of cell markers. **(D)** Percent tumor genotype graph for each cluster. Clusters are ordered by identified tumor cells. MAC and T-cell populations were generally decreased in DN-Wnt1 and K8iKOR-Wnt1 tumors. (MACs = monocytes/macrophages, TC = T cells, FIBs = fibroblasts, EPI = epithelial cells, EC = endothelial cells).

**Figure 4:**
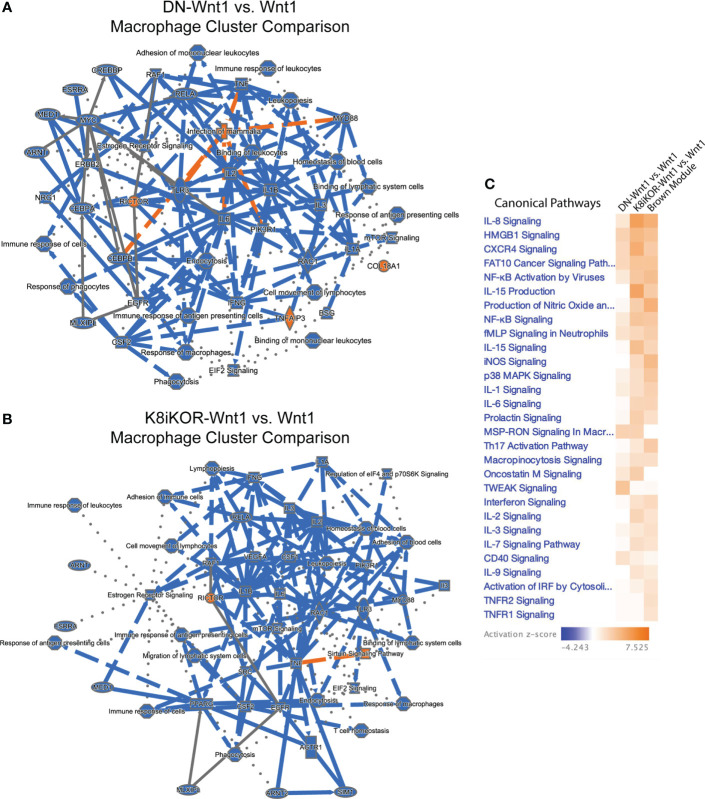
** (A, B)** Macrophage and immune signaling pathways are altered with reduced IGF1R. IPA graphical summary of top pathway alterations in DN-Wnt1 **(A)** or K8iKOR-Wnt1 **(B)** compared to Wnt1 tumors from Cluster 2 (MACs). Blue=downregulated; orange=upregulated. **(C)** IPA canonical pathways heat map of DN-Wnt1 and K8iKOR-Wnt1 compared to Wnt1 tumors and the METABRIC brown (immune signaling signature) module.

Reduced IGF1R by function or expression results in increased tumor metastasis in the mouse models and aligns with human survival data indicating an inverse relationship between IGF1R expression and overall patient survival (17). The mechanisms by which IGF1R regulates tumor metastasis could include intrinsic epithelial mesenchymal transition (EMT) changes as well as alterations to the tumor microenvironment (TME) secondary to the genetic changes in the tumor epithelium. To reveal underlying mechanisms and cell population changes downstream of alterations in IGF1R, we performed single cell RNA-sequencing (scRNA-seq) on the DN-Wnt1, K8iKOR-Wnt1 and Wnt1 tumors. We initially analyzed scRNA-seq of the whole tumor to profile changes in tumor cell populations when IGF1R is either reduced or attenuated in the tumor epithelium. Wnt1 control, DN-Wnt1 and K8iKOR-Wnt1 tumor cells were plotted together resulting in 17 separate tumor cell populations (**Figure 3A**). These populations were further defined using cell specific markers resulting in the following distinct cell populations: 7 epithelial, 2 fibroblast (FIBs), 6 macrophage/monocyte (MACs), 1 T-cell, and 1 endothelial (EC) (**Figures 3B, C**; **Supp. Figure 3**). Overall, loss of IGF1R expression or function resulted in decreased macrophage and T cell populations and expanded fibroblast populations (**Figure 3D**). Furthermore, flow cytometry analysis validated increased fibroblasts (**Supp. Figures 4A–C**) and decreased T cells (17) in tumors with reduced IGF1R function. Ingenuity pathway analysis (IPA) supports the conclusion that loss of IGF1R function promotes an immune evasive TME (**Figures 4A, B**; **Supp. Figure 5**). For example, while the cell number is unchanged in MAC Cluster 2 from DN-Wnt1 and K8iKOR-Wnt1 tumors compared to Wnt1 tumors, the immune function pathways are altered with downregulation of genes involved in immune cell activation, antigen presentation, cell adhesion, and infiltration (**Figures 4A, B**).

Alignment of the immune signature module from the METABRIC data analysis (**Figure 1D**) revealed several immune signaling pathways in human tumors similarly associated with low IGF1R expression as for the mouse tumors with reduced IGF1R function or expression (**Figure 4C**). Interestingly, the pathways upregulated in both patient and mouse tumors with reduced IGF1R are important for response to stress signaling and immune cell evasion supporting our prior findings that loss of IGF1R promotes cell stress in human breast cancer cells (17).

### Expansion of the metastatic tumor epithelial population with reduced IGF1R

**Figure 5:**
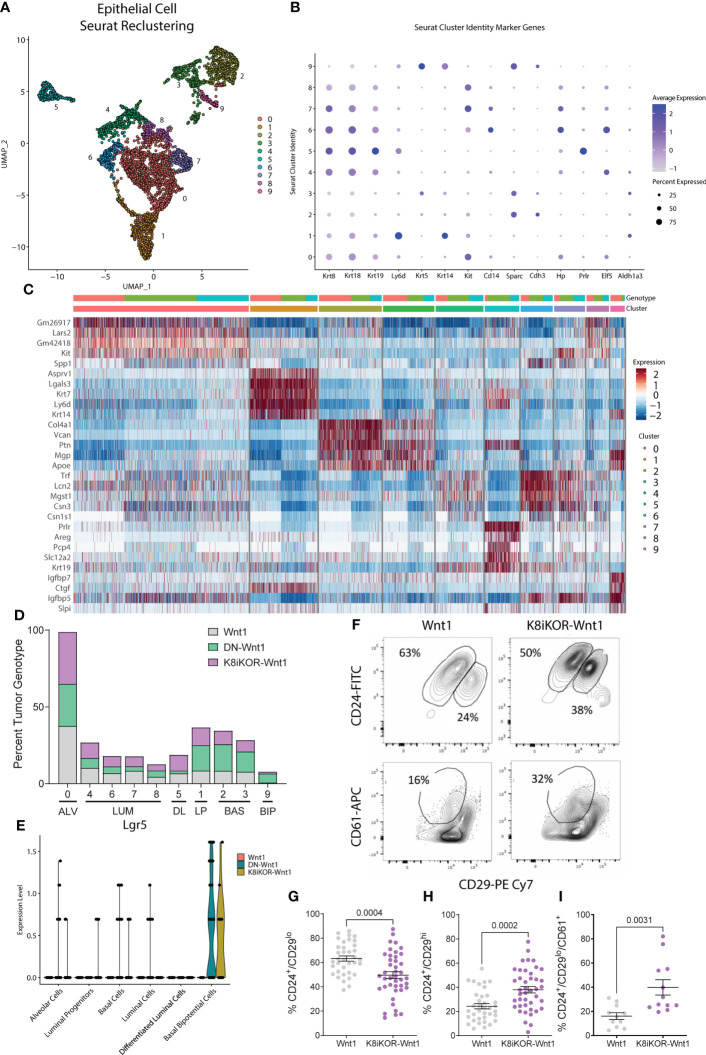
Epithelial cell populations are altered with reduced IGF1R. **(A)** UMAP plot of re-clustering of epithelial cells from Wnt1, DN-Wnt1, and K8iKOR-Wnt1 tumors resulting in 13 clusters. **(B)** Dot plot of epithelial cell markers. **(C)** Heat map of top epithelial cell type markers. Top legend: top row=tumor identity: red=Wnt1, green=DN-Wnt1, blue=K8iKOR-Wnt1; Bottom row=epithelial cell cluster. **(D)** Percent tumor genotype graph for each cell cluster labelled with each cell type defined by markers. (ALV=alveolar cell, LUM=luminal cell, DL=differentiated luminal cell, LP=luminal progenitor, BAS=basal cell, BIP=basal bipotential progenitor). **(E)** Violin plot for Lgr5 in each annotated cluster and tumor type. **(F)** Representative contour plots of flow cytometry of the CD24^+^/CD29^lo^ (luminal) and CD24^+^/CD29^hi^ (basal) cell populations and CD24^+^/CD29^lo^/CD61^-^ (luminal progenitor) cell population in Wnt1 and K8iKOR-Wnt1 tumors. **G-I.** Quantification of luminal **(G)**, basal **(H)**, and luminal progenitor **(I)** populations in Wnt1 and K8iKOR-Wnt1 tumors. Each dot represents an individual tumor. *Statistic:* Unpaired Student’s *t-*test.

We then asked 1) what are the cells from the DN-Wnt1 or K8iKOR-Wnt1 primary tumors that seed lung metastases and 2) what properties of the epithelial cells from the DN-Wnt1 and K8iKOR-Wnt1 tumors promote metastasis? To address these questions, we restricted the scRNA-Seq analysis to the tumor epithelial cell populations. Unsupervised clustering using UMAP resulted in 10 distinct epithelial populations (E0-E9) consisting of 2,543 cells from Wnt1, DN-Wnt1, and K8iKOR-Wnt1 tumors (**Figure 5A**). Using Seurat and heat map analysis of known epithelial cell population markers (37) we identified the epithelial clusters as: alveolar (E0), luminal (E4,E6,E7,E8), differentiated luminal (E5), luminal progenitor (E1) and basal (E2, E3, E9), one of which (E9) had high expression of the bipotential cell marker Lgr5 (**Figures 5B–E**; **Supp. Figure 6**). Importantly, the basal cell clusters (E2,E3), luminal progenitor cluster (E1), and bipotential cluster (E9) were expanded in one or both the K8iKOR-Wnt1 and DN-Wnt1 tumors (**Figure 5D**). The expansion of the basal and luminal progenitor populations in the IGF1R deficient tumors was supported by flow cytometry analyses of the DN-Wnt1 tumors (19) and the K8iKOR-Wnt1 tumors (**Figures 5F–I**). Furthermore, the DN-Wnt1 luminal cells were decreased in each cluster suggesting loss of IGF1R function causes luminal cells to either gain basal markers or to de-differentiate into a more basal phenotype. This is supported by data evaluating K14 expression in sorted tumor luminal cells from tumors with reduced IGF1R (**Supp. Figure 7**). These data revealed an increase in K14 expression in epithelial populations in the IGF1R deficient tumors (**Supp. Figure 7A**) which was seen only in the sorted luminal epithelial population in the DN-Wnt1 tumors compared to Wnt1 tumors (**Supp. Figure 7B**).

**Figure 6:**
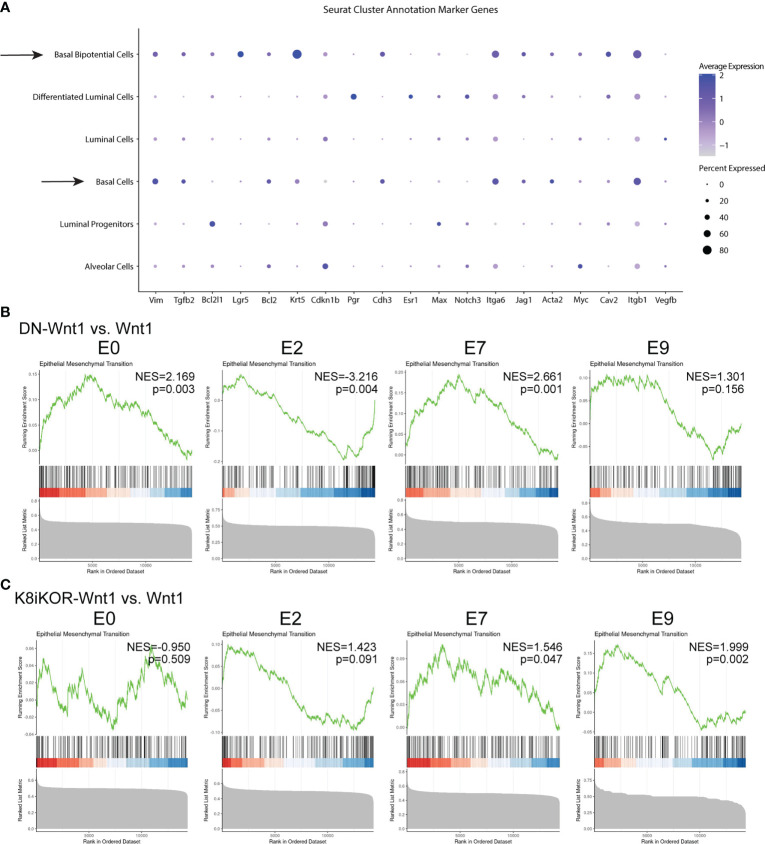
A metastatic and EMT phenotype is enhanced in tumors with reduced IGF1R. **(A)** Dot plot from all tumors of alignment with metastatic signature. Arrows depict clusters with high expression of markers indicating metastatic cell type. **(B, C)** GSEA plots for epithelial mesenchymal transition (EMT) hallmark signature in DN-Wnt1 vs. Wnt1 **(B)** and K8iKOR-Wnt1 vs. Wnt1 **(C)** for luminal clusters E0 and E7, basal cluster E2, and bipotential basal cluster E9. NES = normalized enrichment score. P values for each comparison are shown on each plot. Nominal p-value was calculated using 1000 permutations, with FDR correction.

The bipotential and basal cells are most closely linked to a previously identified metastatic signature (38) (**Figure 6A**). Expansion of the metastatic bipotential and basal populations is consistent with increased metastasis in the IGF1R deficient tumor models (**Figures 2D, E**, **6A**). Gene Set Enrichment Analysis (GSEA) confirmed enrichment in EMT (**Figures 6B, C**; **Supp. Figure 8**) in both the DN-Wnt1 and K8iKOR-Wnt1 tumor epithelial cells, but these analyses began to reveal some distinctions between the two IGF1R deficient tumors. For example, the basal cluster (E2) and the alveolar/luminal clusters (E0, E7) from the DN-Wnt1 tumors showed increased EMT hallmark signature gene expression in the GSEA analysis, whereas the luminal cluster (E7) and bipotential cluster (E9) in the K8iKOR-Wnt1 tumors had the most pronounced GSEA EMT signatures (**Figures 6B, C**). This is also consistent with the enrichment dot plot analyses where the strongest EMT profile is seen in the luminal cluster in the DN-Wnt1 tumors and in the bipotential cluster in the K8iKOR-Wnt1 tumors (**Supp. Figure 8**).

Targeted analysis of the whole tumor using an EMT specific RT2 qPCR assay resulted in increased expression in EMT related genes in DN-Wnt1 tumors compared to Wnt1 tumors (**Supp. Figure 9A**). IPA further revealed key changes in differentiation, cell migration, invasion, and adherence pathways specific to clusters E0, E2, E7, and E9 in the DN-Wnt1 tumors (**Supp. Figures 9B–E**). Increased EMT transcripts (**Figures 6B, C**; **Supp. Figures 8**, **9**) support the conclusion that the epithelial populations are gaining mesenchymal characteristics consistent with increased metastatic potential and increased bipotential populations in the IGF1R deficient tumors.

### Cell adherence is altered in tumor epithelial cells with decreased IGF1R function

**Figure 7:**
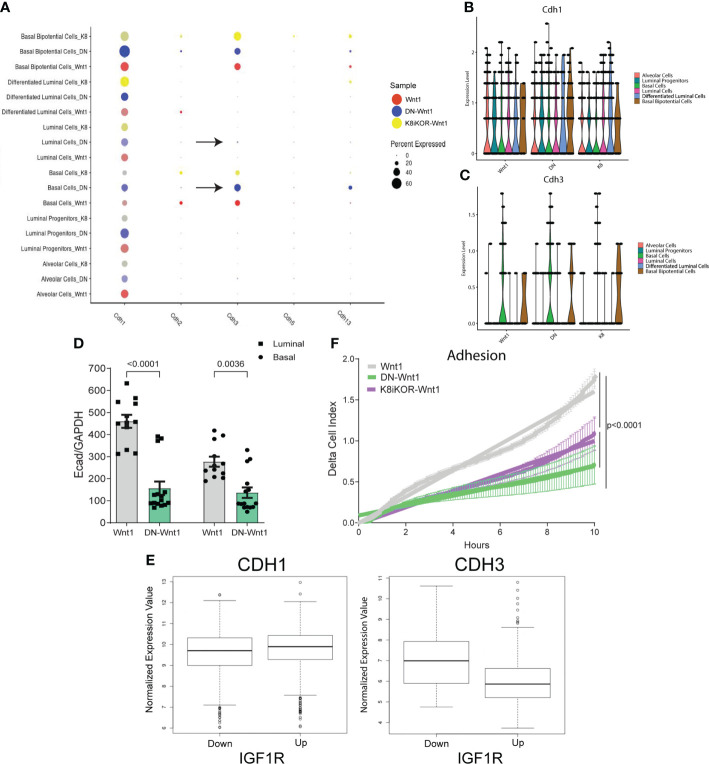
Reduced IGF1R function decreases tumor cell adhesion. **(A)** Dot plot of various cadherins expressed in epithelial tumor cell clusters. Arrows depict clusters with an increase in P-cadherin in the DN-Wnt1 tumors. **(B, C)** E-cadherin **(B)** and P-cadherin **(C)** expression in annotated epithelial cell types identified with single-cell sequencing in Wnt1, DN-Wnt1, or K8iKOR-Wnt1 primary tumors. **(D)** RT-PCR for E-cadherin from Wnt1 or DN-Wnt1 sorted luminal and basal epithelial tumor cells. *Statistic:* Non-parametric Mann-Whitney U test. **(E)** METABRIC data analysis for E-cadherin or P-cadherin in patient tumors with low IGF1R (IGF1R z-score < -1) or high IGF1R (IGF1R z-score > 1) (p < 2.0x10^-16^) *Statistic:* Student’s *t*-test. **(F)** Measurement of adhesion from Wnt1 (grey), DN-Wnt1 (green), or K8iKOR-Wnt1 (purple) by delta cell index over time for 6 hours using the real-time xCELLigence assay. n=3; *Statistic:* Non-linear regression least squares regression for slope best fit p<0.0001 for Wnt1 control compared to DN-Wnt1 or K8iKOR-Wnt1.

Recently, the Ewald lab reported E-cadherin loss is required for metastatic invasion, and its re-expression is necessary to promote metastatic growth (39). To determine whether cadherin expression is altered in tumors with reduced IGF1R, we screened for cadherin expression in each epithelial cluster from the scRNA-Seq data. As expected, luminal cell types had higher E-cadherin (Cdh1) expression whereas basal cell types had higher P-cadherin (Cdh3) and T-cadherin (Cdh13) expression (**Figure 7A**). Interestingly, bipotential cells have high expression of E-cadherin, as well as P-cadherin (**Figures 7A–C**) suggesting a less differentiated cell type. Notably, tumor epithelium with reduced IGF1R resulted in increased P-cadherin expression in DN-Wnt1 and K8iKOR-Wnt1 bipotential cells (**Figures 7B–C**). Furthermore, E-cadherin expression was reduced in both luminal and basal lineages in sorted DN-Wnt1 tumor epithelial cells compared to Wnt1 cells (**Figure 7D**). To determine if cadherin expression similarly changes with IGF1R expression in patient tumors, we analyzed the METABRIC dataset and identified a positive correlation of E-cadherin with IGF1R expression but an inverse correlation of P-cadherin and IGF1R expression across all breast tumors (**Figure 7E**).

To test the functional role of adherence gene changes, we measured tumor epithelial cell adherence *in vitro*. Adherence was decreased in DN-Wnt1 and K8iKOR-Wnt1 compared to Wnt1 primary tumor epithelial cells *in vitro* (**Figure 7F**). Consistent with these findings, DN-Wnt1 primary tumor epithelial cell clusters and single tumor epithelial cells had decreased adherence to collagen matrix compared to Wnt1 primary tumor cells (**Supp. Figures 10A–F**). In contrast, there was no significant difference between the K8iKOR-Wnt1 and Wnt1 primary tumor epithelial cells in their ability to adhere to collagen (**Supp. Figures 10A–F**). Immunofluorescence revealed increased K14^+^ and decreased K8+ cell adherence from DN-Wnt1 compared to Wnt1 primary tumors both in clusters and individual cells (**Supp. Figures10F, G**). Moreover, the non-adherent cells from the DN-Wnt1 tumors had increased E-cadherin expression indicating it was the luminal epithelial cells with reduced IGF1R signaling that had an adherence deficiency (**Supp. Figure 10H**). Furthermore, adherent DN-Wnt1 tumor epithelial cells had increased vimentin suggesting mostly basal cell adhesion with reduced IGF1R. These findings support the hypothesis that disruption of IGF1R in both the luminal and basal lineages in the DN-Wnt1 tumors (see below) may be necessary to disrupt adhesion between epithelial cells. These data support changes in adhesion to substrate but without an effect on cell survival. It is also interesting that in our prior study we showed that Wnt1 tumor epithelial cells increase tumorsphere formation frequency in non-adherent conditions after IGF1R inhibition (19).

Although the two IGF1R deficient models are similar in having elevated metastases and increased basal and EMT phenotypes in the epithelial cells (**Figures 6B, C**, **Supp. Figures 7**,**8**), they also show some differences particularly in cell adherence phenotypes (**Figure 7F**, **Supp. Figure 10**). Two possible explanations for the discrepancy in the adherence phenotype and gene expression pathway alterations between the two models are the mode and lineage specificity of IGF1R disruption. The DN-Wnt1 model expresses a dominant-negative IGF1R transgene that inhibits IGF1R tyrosine kinase function. In this model, the MMTV promoter is active early in the mammary epithelial lineage such that both lineages express the transgene (40). RNAscope immunofluorescence analysis for the human *dnIGF1R* transgene confirmed expression in hyperplastic mammary glands and tumors from the DN-Wnt1 mice (**Supp. Figures 11A–H**). We further verified the expression of the *dnIGF1R* transgene in both luminal and basal epithelial lineages by performing qRT-PCR for the human *dnIGF1R* transgene in tumor epithelial cells following FACS (**Supp. Figure 11I**). In contrast to the DN-Wnt1 model, the K8iKOR-Wnt1 model has an *Igf1r* gene deletion specifically in the K8 luminal lineage. Thus, disruption of receptor signaling versus complete loss of the receptor could lead to different phenotypes as well as the disruption of the IGF1R in both epithelial lineages compared to the luminal lineage only.

### Cell adherence is dysregulated by enhanced P-cadherin expression in epithelial cells with reduced IGF1R function

**Figure 8:**
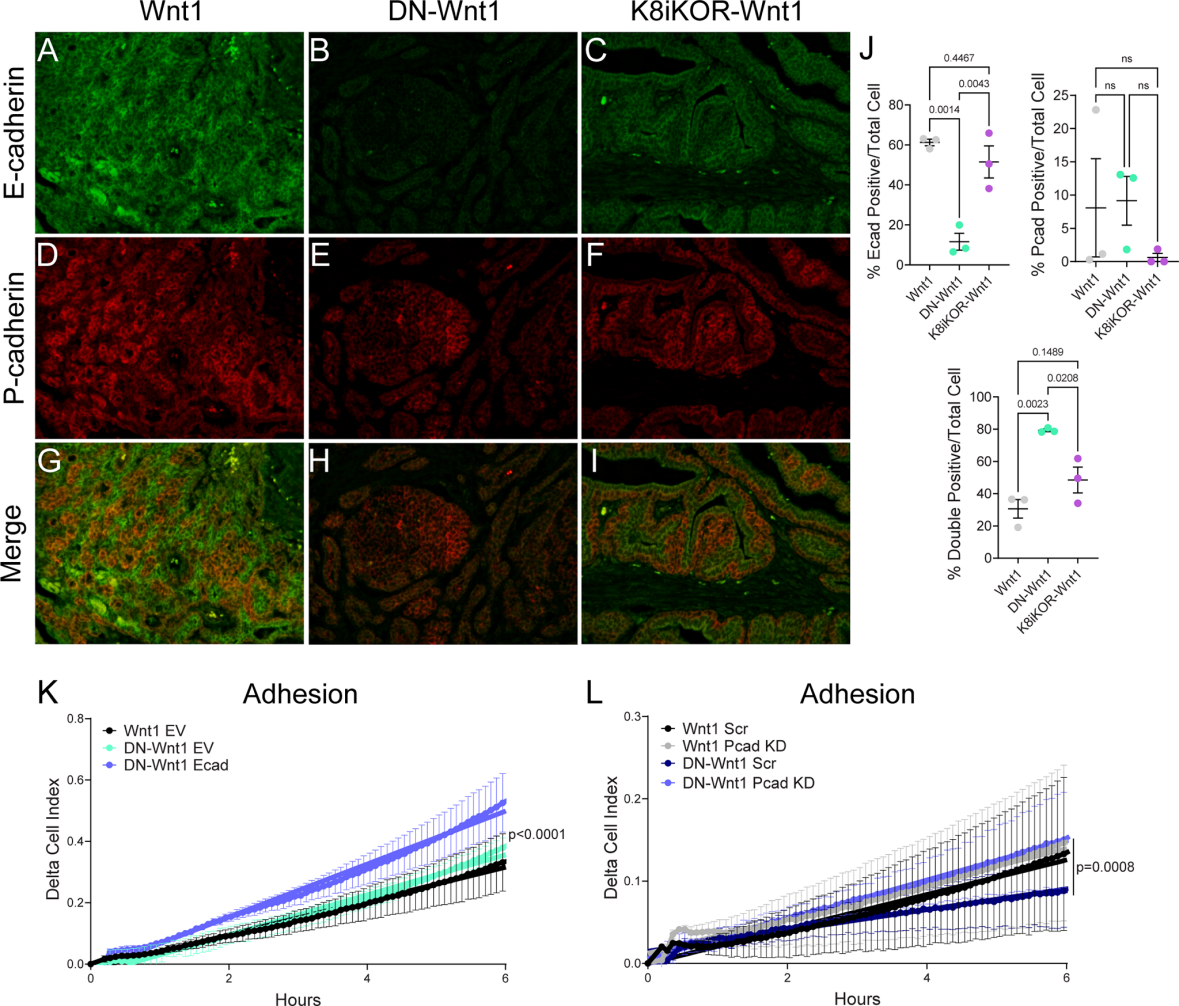
Altered cadherin expression in tumors with reduced IGF1R. **(A–I)** Representative images of E-cadherin (green) or P-cadherin (red) immunostaining in Wnt1 **(A, D, G)**, DN-Wnt1 **(B, E, H)**, and K8iKOR-Wnt1 **(C, F, I)** primary tumors. **(J)** E-cadherin, P-cadherin, and double positive cell count graphs of primary tumors. *Statistic:* One-Way ANOVA with Tukey’s Multiple Comparison *post-hoc* test. **K.** Adhesion (delta cell index) over time in Wnt1 or DN-Wnt1 primary tumors with empty vector (EV) or E-cadherin overexpression (Ecad). n = 3; *Statistic:* Non-linear regression. **(L)** Adhesion (delta cell index) over time in Wnt1 or DN-Wnt1 with P-cadherin knockdown (Pcad KD). n = 3; *Statistic:* Non-linear regression.

Since cadherin gene expression levels are altered with reduced IGF1R, we further analyzed protein levels in tumor tissues to correlate with gene expression. Immunostaining of tumors showed decreased E-cadherin and increased P-cadherin protein expression in DN-Wnt1 and K8iKOR-Wnt1 primary tumors compared to Wnt1 tumors (**Figures 8A–J**). Interestingly, total E-cadherin expression was altered primarily at the protein level in the DN-Wnt1 tumors. Importantly, co-expression of E-cadherin and P-cadherin was increased in DN-Wnt1 and K8iKOR-Wnt1 tumors (**Figures 8G–J**). Co-expression of P-cadherin with E-cadherin in the primary tumor is a marker of more aggressive, metastatic breast tumors (41–44). Thus, reduced IGF1R was associated with altered E-cadherin and P-cadherin in tumor epithelial cells.

To test the functional role of altered E-cadherin and P-cadherin in cells with attenuated IGF1R, we first transiently re-expressed E-cadherin in DN-Wnt1 primary tumor epithelial cells and measured cell adhesion *in vitro*. Overexpression of E-cadherin increased epithelial cell adhesion compared to empty vector control (**Figure 8K**). Furthermore, reducing P-cadherin in DN-Wnt1 primary tumor epithelial cells significantly increased tumor adhesion restoring adhesion back to the level of the Wnt1 tumor cells (**Figure 8L**). Thus, altering cadherins in DN-Wnt1 primary tumor epithelial cells rescues the compromised adherence suggesting these changes in E- and P-cadherins due to reduced IGF1R are necessary for metastasis.

## Discussion

A major question in cancer biology is how do primary tumor cells metastasize to another site? Here we show loss of IGF1R in the primary tumor promotes metastasis by modulating cadherin expression and altering epithelial cell properties to decrease cellular adhesion. While it is well established that epithelial cells gain mesenchymal cell properties to migrate out of the primary tumor (45–49), several recent studies have shown only a subset of mesenchymal properties are necessary for migration and invasion referred to as partial EMT (50–53). While original dogma was that the metastatic process occurs by single tumor epithelial cell migration and invasion, recent observations of collective epithelial cell migration have presented a new mechanism for metastasis that relies on interactions between a mesenchymal-like leader cell with other epithelial cells in the primary tumor (45). Thus, understanding how cell-cell interactions are regulated both in the primary tumor and at distant sites of colonization is critical to determining metastatic potential of tumor cells.

Loss of E-cadherin is a hallmark of EMT and necessary for basal cells to adapt to becoming leader metastatic cells (46). The Ewald lab previously described a process by which the transition of E-cadherin expression is critical for collective invasion (39). Here, we have shown E-cadherin expression is decreased in mouse models with reduced function or expression of IGF1R to drive collective invasion. Prior reports have also linked E-cadherin and IGF1R in breast cancers. Proteomic screening and network analyses of breast cancer cell lines stimulated with either IGF-1 or insulin suggested signaling interactions between the two pathways (54). In subsequent validation of these analyses, the authors demonstrated that knockdown of E-cadherin augmented p-Akt levels particularly in cells stimulated with IGF-1 (54). A subsequent report showed direct interaction between IGF1R and E-cadherin and similarly showed that loss of E-cadherin increased activation of IGF1R signaling (55). Our data seemingly contradict these findings; however, our studies analyzed effects on E-cadherin and adhesion from the perspective of IGF1R reduction rather than the reverse. It is possible that the interaction between the two proteins helps stabilize E-cadherin but also suppresses IGF1R signaling. Our data also reveal that attenuation or reduced IGF1R in the Wnt1-driven tumors augments P-cadherin expression. Interestingly, recent reports have shown acquisition of P-cadherin is necessary for tumor cells to become metastatic. More importantly, the co-expression of P-cadherin and E-cadherin is critical for enhanced metastasis and suggests these cells are exhibiting a partial EMT phenotype. The co-expression of P-cadherin and E-cadherin and a partial EMT phenotype in IGF1R-reduced Wnt1 tumors suggests increased metastatic properties of these tumor cells.

While loss of IGF1R is sufficient to drive a partial EMT phenotype and collective invasion to promote metastasis, alterations in the tumor microenvironment may also be required for increased tumor extravasation. Our previous studies showed heightened cell stress driven by attenuated IGF1R resulted in immune cell evasion and a pro-metastatic tumor microenvironment (17). Single-cell RNA sequencing analysis of tumors with reduced IGF1R recapitulate these previous data by showing depletion of immune cell populations and alterations in immune cell function genes and pathways. Furthermore, stroma changes shown in our previous study (17) could be attributed to expansion of fibroblast populations in tumors with reduced IGF1R function (**Figure 3D**; **Supp. Figure 4**). Taken together, it is clear that loss of IGF1R in mammary tumors alters the microenvironment to promote metastasis.

One question that arises from inhibiting IGF1R in our tumor models is whether there may be compensatory expression or activation of the insulin receptor (INSR). In our initial publication on development of the DN-Wnt1 tumor model (19), we found that P/T Akt and P/T Erk were reduced in normal mammary epithelial cells expressing the DN-IGF1R. Moreover, P/T IRS-1 was decreased in the DN-Wnt1 tumors compared to Wnt1 tumors. These data argue against compensation by increased INSR signaling. However, we did see a shift in the *Insr-A:Insr-B* isoform ratio as well as increased expression of *Igf2* mRNA in the DN-Wnt1 tumors supporting an IGF-II/INSR-A signaling loop. From the scSeq data in the current analyses, we observed a reduction in *Insr* mRNA expression in luminal clusters 5,7 and 8 in both the DN-Wnt1 and K8iKOR-Wnt1 tumors compared to Wnt1 tumors. This was confirmed by RT-PCR analyses (not shown). However, western blot analyses of total INSR expression indicated no significant change in INSR at the protein level (analyzed in DN-Wnt1 tumors vs Wnt1 tumors; not shown). Interestingly, TCGA analysis of human breast tumors revealed a positive correlation between IGF1R and INSR expression (56). Thus, while we cannot entirely rule out an increase in INSR activation in the IGF1R deficient tumor models, there is not a compensatory increase in expression of the INSR.

While a similar metastatic process is observed in the DN-Wnt1 and K8iKOR-Wnt1 primary tumor models, the scRNA-seq analysis revealed clear differences in the genomic profile of the primary tumor cells in these models. Similarly, minor phenotypic differences have been observed when measuring cell adherence. There are two key differences in these models that likely contribute to these findings: 1) the DN-Wnt1 model attenuates the receptor activity whereas the K8iKOR-Wnt1 model is a gene knockout in the luminal epithelium, and 2) the *dnIGF1R* transgene is expressed in luminal and basal epithelial cells blocking the receptor function in all mammary epithelium, whereas receptor expression is decreased only in the luminal epithelial cells in the K8iKOR-Wnt1 model leaving the basal cell IGF1R intact. Potentially, the loss of IGF1R function in both luminal and basal epithelial cells may lead to the heightened model phenotype because of reduced adherence. These findings emphasize modeling importance.

**Figure 9:**
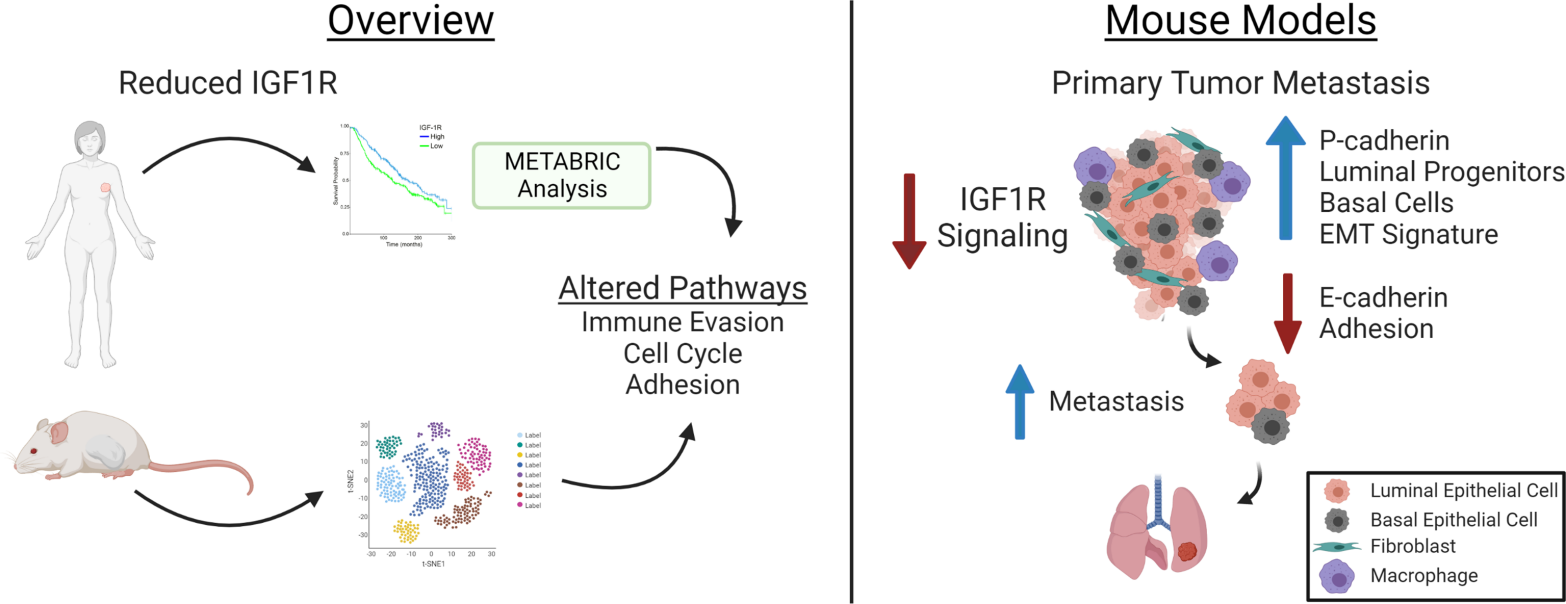
Model for how Reduced IGF1R in Human Breast Tumors (left) and in Mouse Basal-Like Mammary Tumors results in Enhanced Metastasis. The overview panel (left) summarizes human breast cancer data showing inverse correlation between IGF1R expression and patient survival and lymph node positivity [see (9, 17)] and from METABRIC data analyses in the current manuscript showing low tumor IGF1R expression is correlated with gene expression indicating increased tumor cell invasion properties and cell cycle and decreased cell adhesion. Similar pathways were identified from scRNA-Seq of the tumors in the mouse models with reduced IGF1R signaling or expression. Analyses of the mouse models (right) revealed decreased IGF1R signaling in primary tumor epithelium resulted in increased lung metastases and alterations associated with metastasis including increased P-cadherin in E-cadherin-positive cells, increased EMT signatures in luminal and basal cell populations and in decreased E-cadherin and cell adhesion. Created with BioRender.com.

It is clear from the spontaneous tumor models attenuated or loss of IGF1R decreases tumor latency and increases metastasis. These results are consistent with the clinical data where trials inhibiting IGF1R have been unsuccessful. The interconnectedness of the tumor epithelium and microenvironment is highly complex. The advantage of our models is the ability to study stochastic tumor progression in the context of the microenvironment which reveals this complex tumor biology. Importantly, the mouse modeling data aligns with the human gene expression and pathway analyses (**Figure 9**) and provides a basis for understanding why loss of IGF1R in human breast cancers is associated with a worse outcome.

## Data availability statement

The datasets presented in this study can be found in online repositories. The names of the repository/repositories and accession number(s) can be found on: https://www.ncbi.nlm.nih.gov/geo/, GSE182236 and on https://osf.io/9mkc4/.

## Ethics statement

The animal study was reviewed and approved by Rutgers University IACUC.

## Author contributions

AO performed the majority of the experiments and statistical analyses, participated in the study design and wrote the manuscript. JB performed metastases quantification, qRT-PCR for *Igf1r* deletion in sorted cell populations, participated in the *in vitro* adhesion assays and study design and in manuscript and figure revisions. Y-JC performed the WGCNA METABRIC analysis. VC performed the initial analyses on the K8iKOR-Wnt1 mouse tumor line. AL performed the scRNA-Seq analyses. KM performed metastases quantification, RNAScope and participated in the *in vitro* adhesion assays and study design. QS performed mouse genotyping, tamoxifen tests, gland analyses and tumor harvesting. EJG and DL contributed to results interpretation and manuscript editing. TLW is the principal investigator for this project and was involved in study design, data analysis, manuscript editing and submission. All authors contributed to the article and approved the submitted version.
