# Supplementary material for: Breast tumor IGF1R regulates cell adhesion and metastasis: alignment of mouse single cell and human breast cancer transcriptomics
Source: Front Oncol. 2022 Dec 7;12:990398. doi: 10.3389/fonc.2022.990398 (PMC9769962; doi:10.3389/fonc.2022.990398)
Supplement: Supplementary file 1 [file DataSheet_1.pdf]

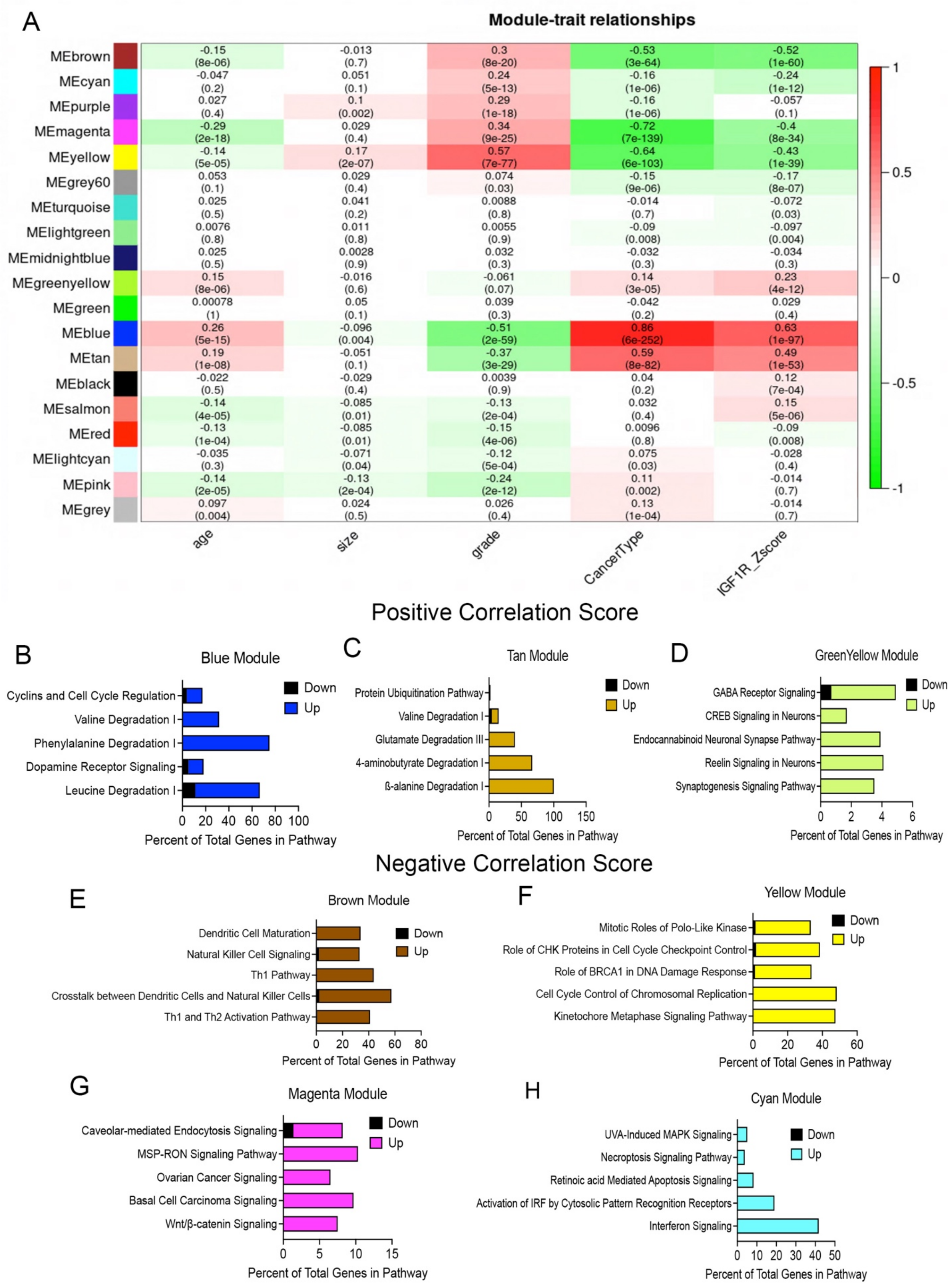

A
Module-trait relationships
MEbrown -0.15 (8e-06) -0.013 (0.7) 0.3 (8e-20) -0.53 (3e-64) -0.52 (1e-60)
MEcyan -0.047 (0.2) 0.051 (0.1) 0.24 (5e-13) -0.16 (1e-06) -0.24 (1e-12)
MEpurple 0.027 (0.4) 0.1 (0.002) 0.29 (1e-18) -0.16 (1e-06) -0.057 (0.1)
MEmagenta -0.29 (2e-18) 0.029 (0.4) 0.34 (9e-25) -0.72 (7e-139) -0.4 (8e-34)
MEyellow -0.14 (5e-05) 0.17 (2e-07) 0.57 (7e-77) -0.64 (6e-103) -0.43 (1e-39)
MEgrey60 0.053 (0.1) 0.029 (0.4) 0.074 (0.03) -0.15 (9e-06) -0.17 (8e-07)
MEturquoise 0.025 (0.5) 0.041 (0.2) 0.0088 (0.8) -0.014 (0.7) -0.072 (0.03)
MElightgreen 0.0076 (0.8) 0.011 (0.8) 0.0055 (0.9) -0.09 (0.008) -0.097 (0.004)
MEmidnightblue 0.025 (0.5) 0.0028 (0.9) 0.032 (0.3) -0.032 (0.3) -0.034 (0.3)
MEgreenyellow 0.15 (8e-06) -0.016 (0.6) -0.061 (0.07) 0.14 (3e-05) 0.23 (4e-12)
MEgreen 0.00078 (1) 0.05 (0.1) 0.039 (0.3) -0.042 (0.2) 0.029 (0.4)
MEblue 0.26 (5e-15) -0.096 (0.004) -0.51 (2e-59) 0.86 (6e-252) 0.63 (1e-97)
MEtan 0.19 (1e-08) -0.051 (0.1) -0.37 (3e-29) 0.59 (8e-82) 0.49 (1e-53)
MEblack -0.022 (0.5) -0.029 (0.4) 0.0039 (0.9) 0.04 (0.2) 0.12 (7e-04)
MEsalmon -0.14 (4e-05) -0.085 (0.01) -0.13 (2e-04) 0.032 (0.4) 0.15 (5e-06)
MEred -0.13 (1e-04) -0.085 (0.01) -0.15 (4e-06) 0.0096 (0.8) -0.09 (0.008)
MElightcyan -0.035 (0.3) -0.071 (0.04) -0.12 (5e-04) 0.075 (0.03) -0.028 (0.4)
MEpink -0.14 (2e-05) -0.13 (2e-04) -0.24 (2e-12) 0.11 (0.002) -0.014 (0.7)
MEgrey 0.097 (0.004) 0.024 (0.5) 0.026 (0.4) 0.13 (1e-04) -0.014 (0.7)
age
size
grade
CancerType
IGF1R_Zscore
1
0.5
0
-0.5
-1
Positive Correlation Score
B
Blue Module
Cyclins and Cell Cycle Regulation
Valine Degradation I
Phenylalanine Degradation I
Dopamine Receptor Signaling
Leucine Degradation I
Down
Up
0 20 40 60 80 100
Percent of Total Genes in Pathway
C
Tan Module
Protein Ubiquitination Pathway
Valine Degradation I
Glutamate Degradation III
4-aminobutyrate Degradation I
ß-alanine Degradation I
Down
Up
0 50 100 150
Percent of Total Genes in Pathway
D
GreenYellow Module
GABA Receptor Signaling
CREB Signaling in Neurons
Endocannabinoid Neuronal Synapse Pathway
Reelin Signaling in Neurons
Synaptogenesis Signaling Pathway
Down
Up
0 2 4 6
Percent of Total Genes in Pathway
Negative Correlation Score
E
Brown Module
Dendritic Cell Maturation
Natural Killer Cell Signaling
Th1 Pathway
Crosstalk between Dendritic Cells and Natural Killer Cells
Th1 and Th2 Activation Pathway
Down
Up
0 20 40 60 80
Percent of Total Genes in Pathway
F
Yellow Module
Mitotic Roles of Polo-Like Kinase
Role of CHK Proteins in Cell Cycle Checkpoint Control
Role of BRCA1 in DNA Damage Response
Cell Cycle Control of Chromosomal Replication
Kinetochore Metaphase Signaling Pathway
Down
Up
0 20 40 60
Percent of Total Genes in Pathway
G
Magenta Module
Caveolar-mediated Endocytosis Signaling
MSP-RON Signaling Pathway
Ovarian Cancer Signaling
Basal Cell Carcinoma Signaling
Wnt/β-catenin Signaling
Down
Up
0 5 10 15
Percent of Total Genes in Pathway
H
Cyan Module
UVA-Induced MAPK Signaling
Necroptosis Signaling Pathway
Retinoic acid Mediated Apoptosis Signaling
Activation of IRF by Cytosolic Pattern Recognition Receptors
Interferon Signaling
Down
Up
0 10 20 30 40 50
Percent of Total Genes in Pathway

Supp. Figure 1. METABRIC weighted gene co-expression network analysis (WGCNA) for all gene probes associated with low or high IGF1R expression in human breast cancers. A. Table of integrated WGCNA (IGF1R-GS1) showing module and clinical trait association. Each row corresponds to a module by its eigengene (ME), each column to a clinical measurement. Each cell contains the corresponding correlation and p-value (in parentheses). The table is color-coded by correlation according to the color legend. Green < 0 for negative correlation; Red > 0, for positive correlation. **B-D.** Top 5 pathways identified by ingenuity pathway analysis (IPA) of selected gene modules from (A) revealing functional signatures in 3 modules positively correlated with IGF1R expression. (blue and tan=metabolic signatures, greenyellow=neuron signature) **E-H.** Top 5 pathways identified by IPA analysis of selected gene modules from (A) revealing key signatures in 4 modules inversely (negatively) correlated with IGF1R expression. (brown module=immune signature, yellow module=cell cycle signature, magenta=Wnt signaling signature, cyan=apoptosis signature).

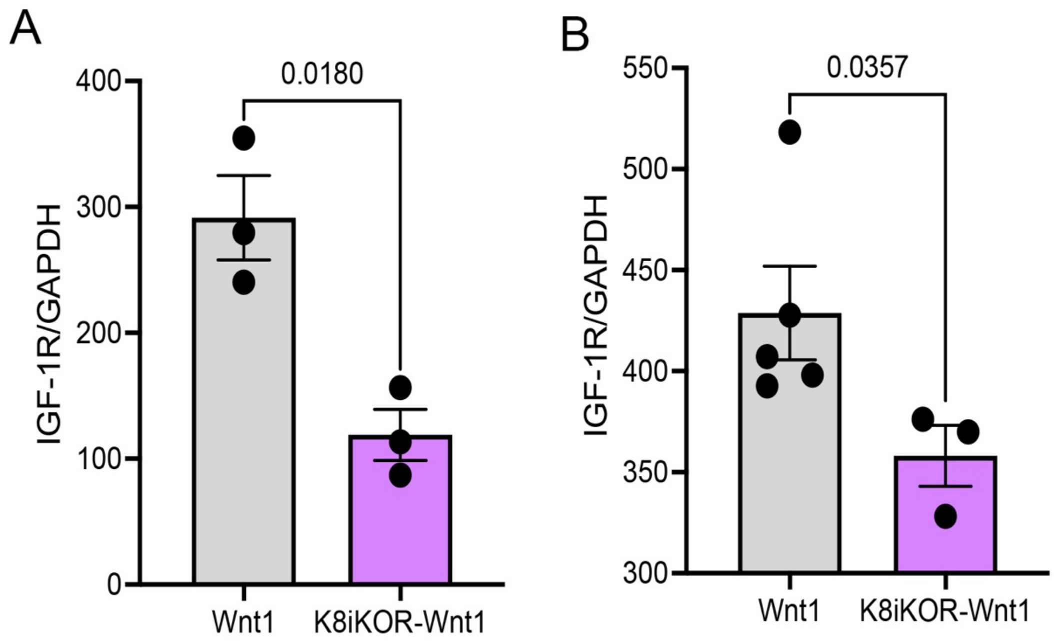

Supp. Figure 2. K8iKOR model validation: *Igf1r* expression in mammary tissue and tumors of K8iKOR-Wnt1 mice. A-B. *Igf1r* expression in 16-week mammary gland hyperplasias **(A)** or end stage tumors **(B)** from Wnt1 or K8iKOR-Wnt1 mice determined by qRT-PCR**.** Expression of *Gapdh* was used for normalization. *Statistic:* Unpaired parametric Welch's *t* test (hyperplasia), Unpaired non-parametric Mann-Whitney U test.

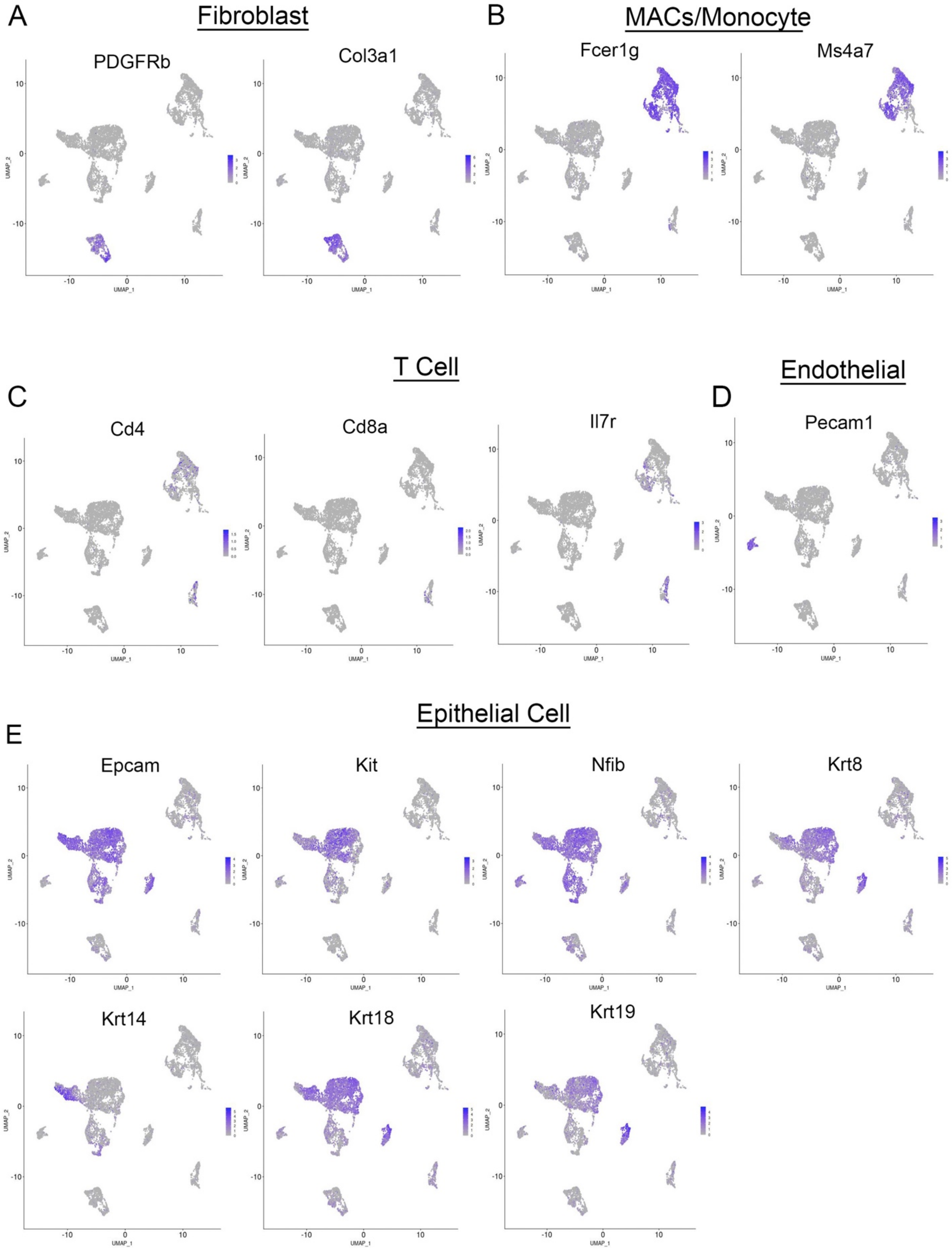

A
Fibroblast
PDGFRb
Col3a1
B
MACs/Monocyte
Fcer1g
Ms4a7
T Cell
C
Cd4
Cd8a
Il7r
Endothelial
D
Pecam1
Epithelial Cell
E
Epcam
Kit
Nfib
Krt8
Krt14
Krt18
Krt19
UMAP_1
UMAP_2

Supp. Figure 3. Identification of tumor cell populations.

A-E. Feature plots from Seurat analysis for cell markers for fibroblasts (*Pdgfrb, Col3a1*) **(A)**, MACs/monocytes (*Fcer1g, Ms4a7*) **(B)**, T cells (*Cd4, Cd8, Il17r*) **(C)**, endothelial cells (*Pecam1*) **(D)**, and epithelial cells (*Epcam, Kit, Nfib, Krt8, Krt14, Krt18, Krt19*) **(E)**.

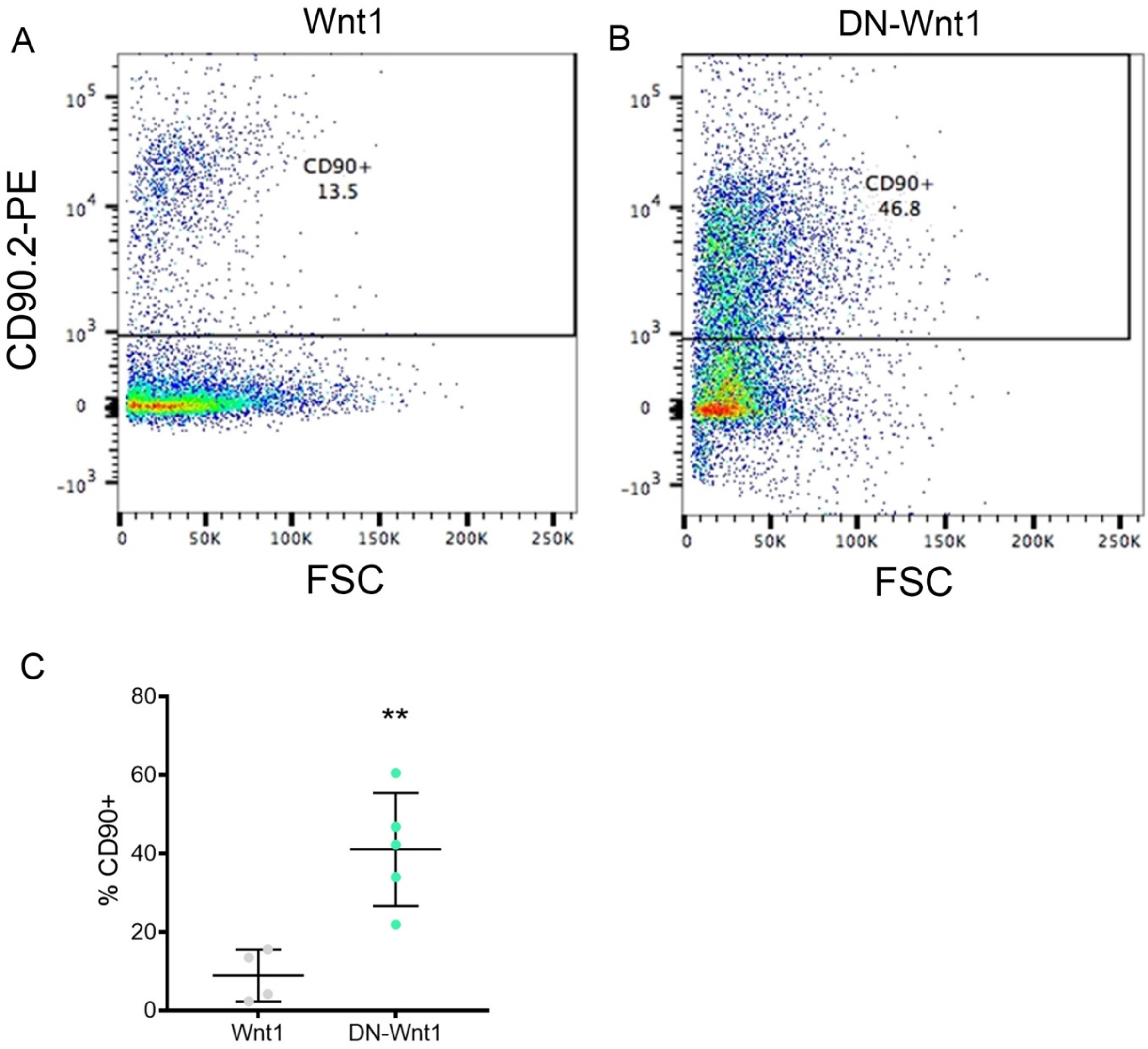

Supp. Figure 4: Increased fibroblasts in tumors with reduced IGF1R function. A-B. Representative dot plots of flow cytometry of the CD90.2 (fibroblast) population in Wnt1 **(A)** and DN-Wnt1 **(B)** tumors. **C.** Quantification of the fibroblast population. Each dot represents an individual tumor. *Statistic:* Unpaired Student's *t*-test

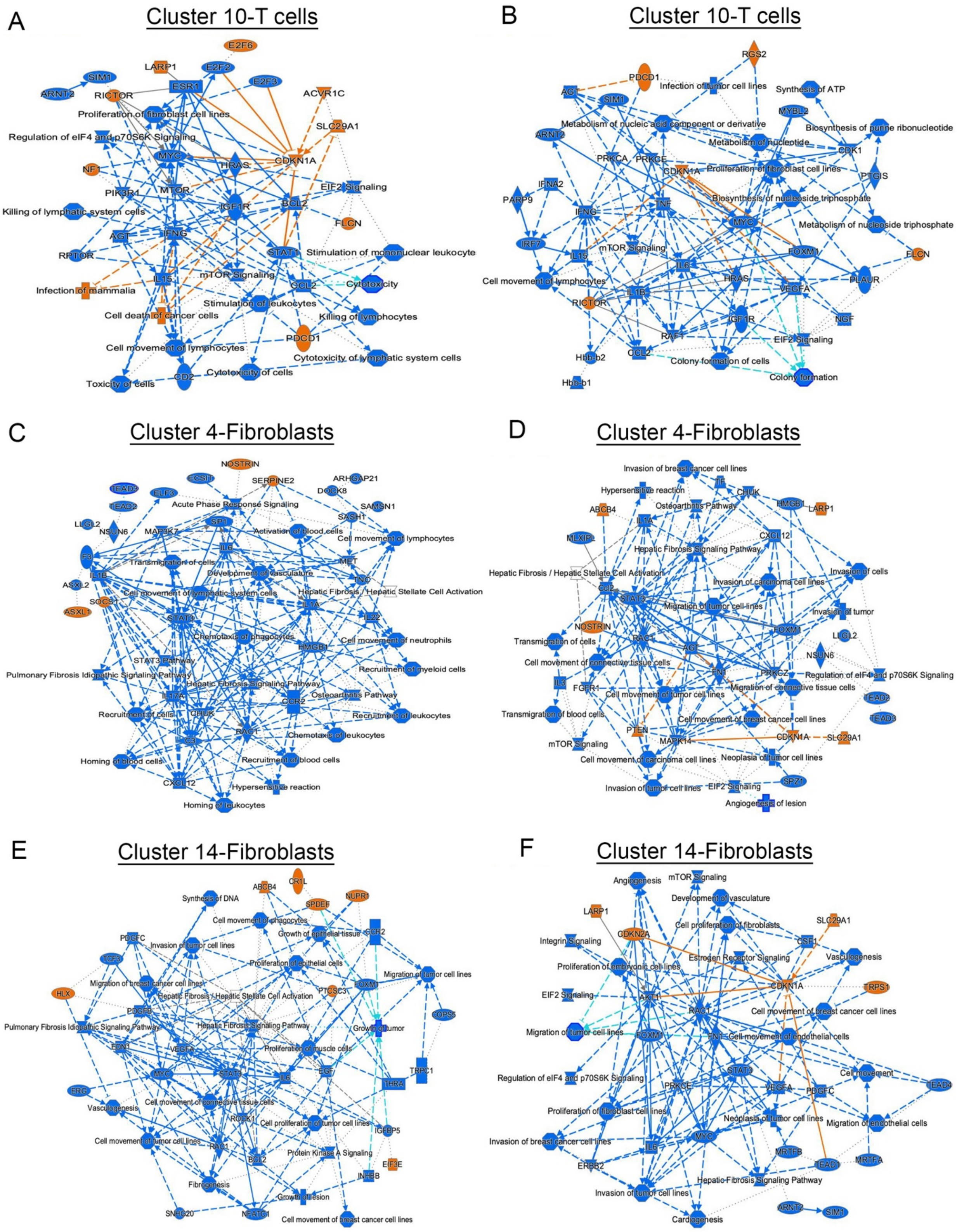

A
Cluster 10-T cells
B
Cluster 10-T cells
C
Cluster 4-Fibroblasts
D
Cluster 4-Fibroblasts
E
Cluster 14-Fibroblasts
F
Cluster 14-Fibroblasts

Supp. Figure 5. Pathway, cell function, and gene changes in tumor cells with reduced IGF1R. A-F. Graphical summary plots for DN-Wnt1 **(A,C,E)** or K8iKOR-Wnt1 **(B,D,F)** vs. Wnt1 tumors for Cluster 10 (T-cells) **(A,B)** and Clusters 4 and 14 (fibroblasts) **(C,D;E,F)**. Blue=downregulated; orange=upregulated.

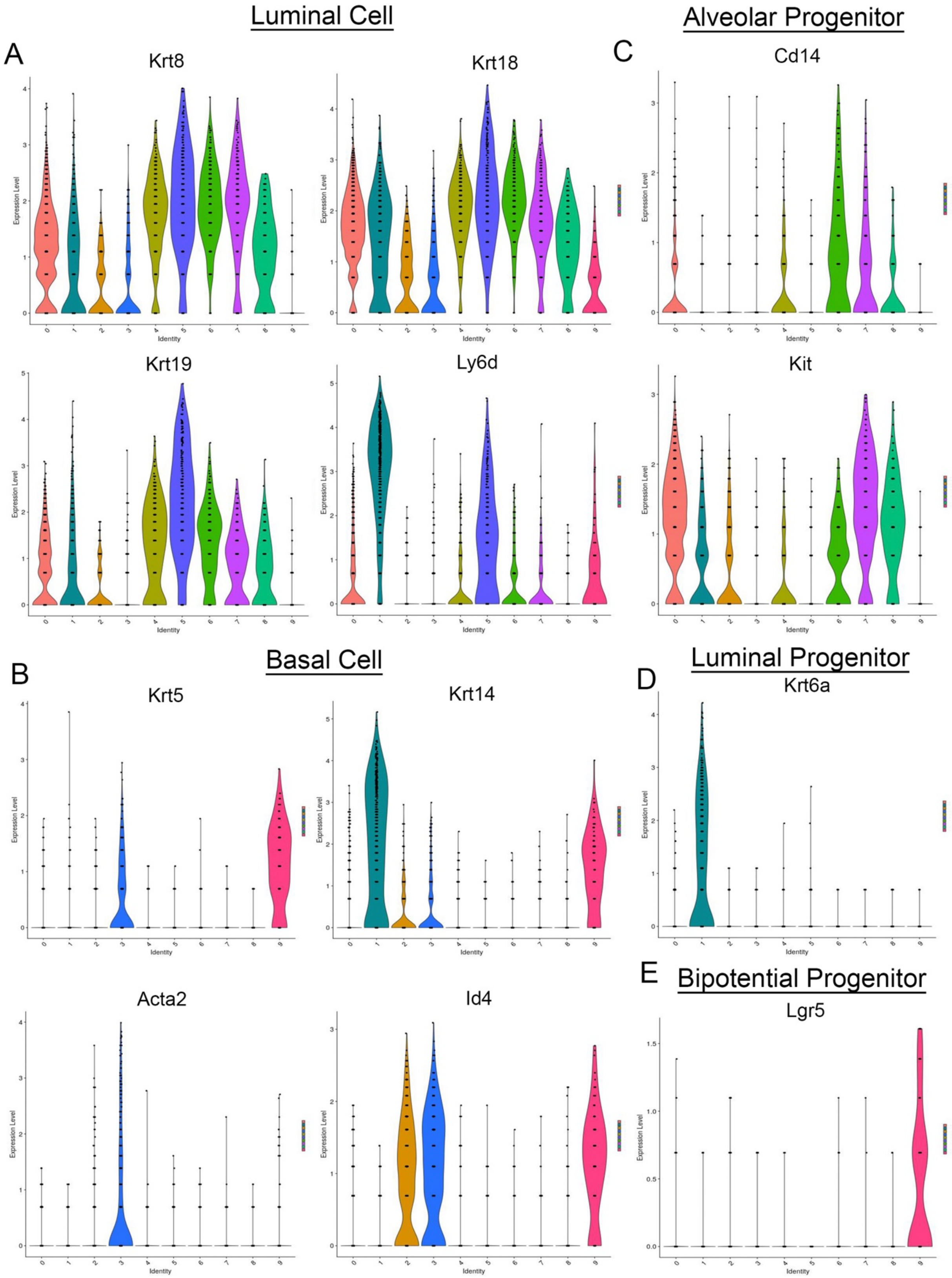
Luminal Cell
A
Krt8
Krt18
Krt19
Ly6d
Alveolar Progenitor
C
Cd14
Kit
Basal Cell
B
Krt5
Krt14
Acta2
Id4
Luminal Progenitor
D
Krt6a
E
Bipotential Progenitor
Lgr5
Expression Level
Identity

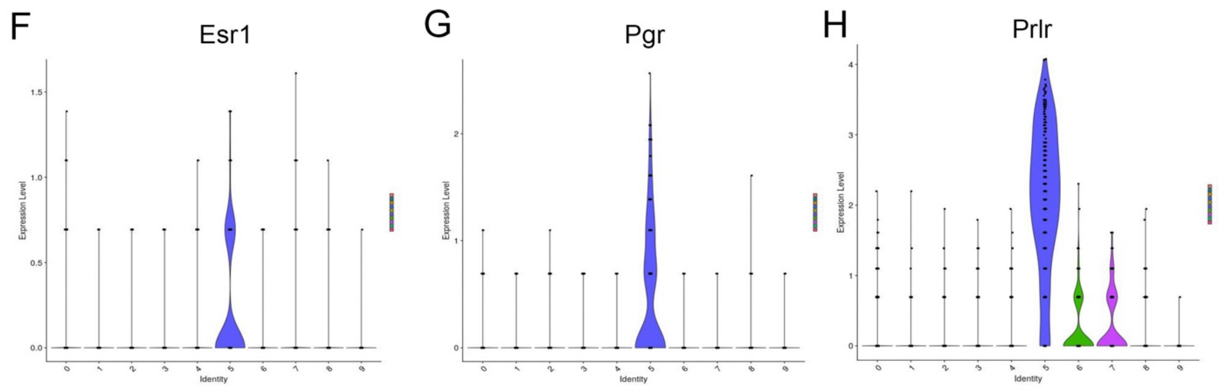

Supp. Figure 6. Epithelial cell subtypes in mammary tumors. A-E. Violin plot for markers for luminal **(A)**, basal **(B)**, alveolar progenitor **(C)**, luminal progenitor **(D)**, and bipotential progenitor cells **(E). F-H.** Cluster 5 has increased levels of estrogen receptor (Esr1) **(F)**, progesterone receptor (Pgr) **(G)**, and prolactin receptor (Prlr) **(H)** identifying this cluster as a hormone-sensing cell.

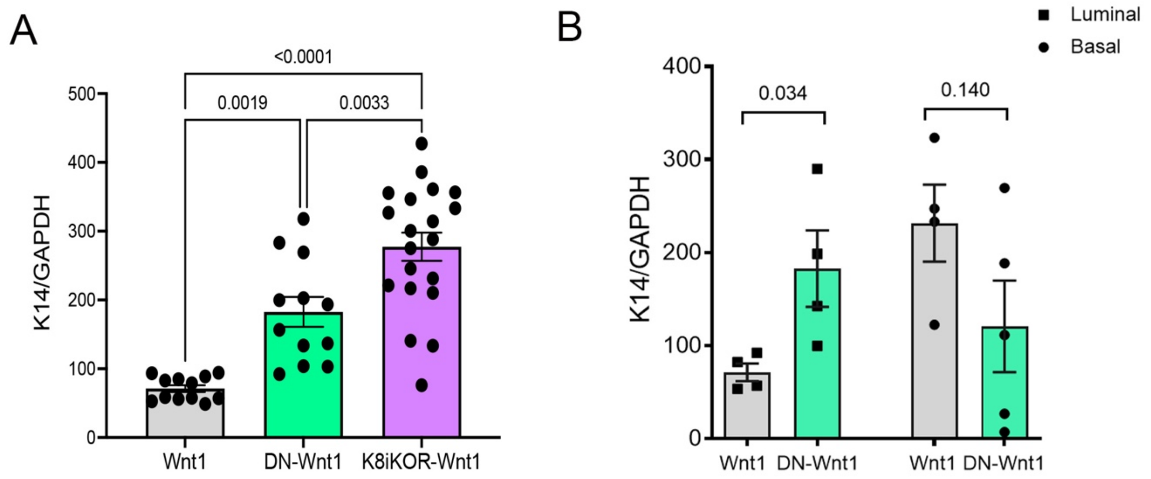

Supp. Figure 7: K14 expression in primary tumor epithelium. A. RT-PCR for K14 epithelial cells from Wnt1, DN-Wnt1, and K8iKOR-Wnt1 tumors. **B.** RT-PCR for K14 in sorted primary tumor cells from Wnt1 and DN-Wnt1 tumors.

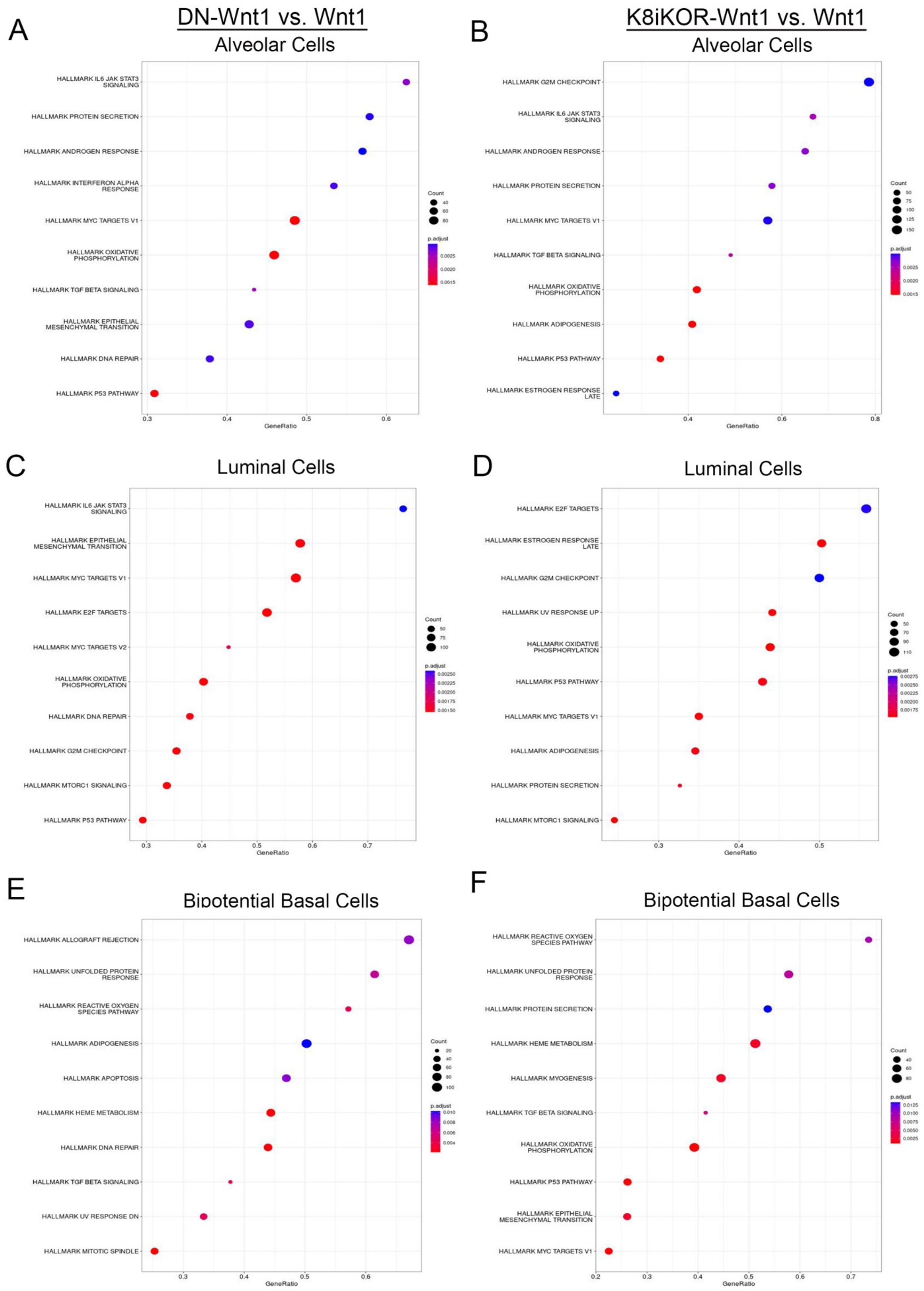

A
DN-Wnt1 vs. Wnt1
Alveolar Cells
HALLMARK IL6 JAK STAT3 SIGNALING
HALLMARK PROTEIN SECRETION
HALLMARK ANDROGEN RESPONSE
HALLMARK INTERFERON ALPHA RESPONSE
HALLMARK MYC TARGETS V1
HALLMARK OXIDATIVE PHOSPHORYLATION
HALLMARK TGF BETA SIGNALING
HALLMARK EPITHELIAL MESENCHYMAL TRANSITION
HALLMARK DNA REPAIR
HALLMARK P53 PATHWAY
GeneRatio
Count
p.adjust
B
K8iKOR-Wnt1 vs. Wnt1
Alveolar Cells
HALLMARK G2M CHECKPOINT
HALLMARK IL6 JAK STAT3 SIGNALING
HALLMARK ANDROGEN RESPONSE
HALLMARK PROTEIN SECRETION
HALLMARK MYC TARGETS V1
HALLMARK TGF BETA SIGNALING
HALLMARK OXIDATIVE PHOSPHORYLATION
HALLMARK ADIPOGENESIS
HALLMARK P53 PATHWAY
HALLMARK ESTROGEN RESPONSE LATE
GeneRatio
Count
p.adjust
C
Luminal Cells
HALLMARK IL6 JAK STAT3 SIGNALING
HALLMARK EPITHELIAL MESENCHYMAL TRANSITION
HALLMARK MYC TARGETS V1
HALLMARK E2F TARGETS
HALLMARK MYC TARGETS V2
HALLMARK OXIDATIVE PHOSPHORYLATION
HALLMARK DNA REPAIR
HALLMARK G2M CHECKPOINT
HALLMARK MTORC1 SIGNALING
HALLMARK P53 PATHWAY
GeneRatio
Count
p.adjust
D
Luminal Cells
HALLMARK E2F TARGETS
HALLMARK ESTROGEN RESPONSE LATE
HALLMARK G2M CHECKPOINT
HALLMARK UV RESPONSE UP
HALLMARK OXIDATIVE PHOSPHORYLATION
HALLMARK P53 PATHWAY
HALLMARK MYC TARGETS V1
HALLMARK ADIPOGENESIS
HALLMARK PROTEIN SECRETION
HALLMARK MTORC1 SIGNALING
GeneRatio
Count
p.adjust
E
Bipotential Basal Cells
HALLMARK ALLOGRAFT REJECTION
HALLMARK UNFOLDED PROTEIN RESPONSE
HALLMARK REACTIVE OXYGEN SPECIES PATHWAY
HALLMARK ADIPOGENESIS
HALLMARK APOPTOSIS
HALLMARK HEME METABOLISM
HALLMARK DNA REPAIR
HALLMARK TGF BETA SIGNALING
HALLMARK UV RESPONSE DN
HALLMARK MITOTIC SPINDLE
GeneRatio
Count
p.adjust
F
Bipotential Basal Cells
HALLMARK REACTIVE OXYGEN SPECIES PATHWAY
HALLMARK UNFOLDED PROTEIN RESPONSE
HALLMARK PROTEIN SECRETION
HALLMARK HEME METABOLISM
HALLMARK MYOGENESIS
HALLMARK TGF BETA SIGNALING
HALLMARK OXIDATIVE PHOSPHORYLATION
HALLMARK P53 PATHWAY
HALLMARK EPITHELIAL MESENCHYMAL TRANSITION
HALLMARK MYC TARGETS V1
GeneRatio
Count
p.adjust

Supp. Figure 8. Epithelial-mesenchymal transition (EMT) is enriched in tumor epithelial cells with reduced IGF1R. A-E. Enrichment dot plots of the gene set enrichment analysis (GSEA) hallmark gene set in DN-Wnt1 **(A,C,E)** and K8iKOR-Wnt1 **(B,D,F)** primary tumors. Alveolar cells **(A)**, luminal cells **(C)**, and bipotential basal cells **(F)** showed enrichment in EMT in tumors with reduced IGF1R.

A

Gene	Fold Change
Mmp3	23.4
Mmp9	10.3
Col3a1	9.8
Fgfbp1	9.1
Twist1	9.1
Fn1	6.6
Col1a2	6.1
Slug	6.1
Pdgfrb	4.9
Wnt11	4.8
Notch1	4.7
Zeb2	4.7

Gene	Fold Change
Mmp2	4.6
Krt14	4.5
Ptk2	4.5
Akt1	4.3
Bmp1	4.3
Itga5	3.8
Col5a2	3.6
Ilk	3.6
Itgav	3.5
Foxc2	3.4
Stat3	3.4
Zeb1	3.2
Vim	3.1

B

DN-Wnt1 E0

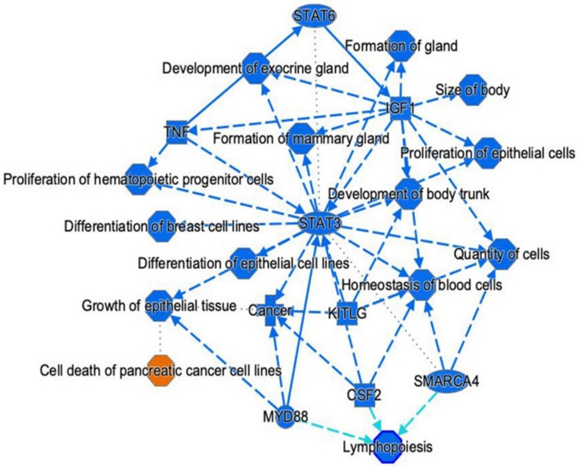

C

DN-Wnt1 E2

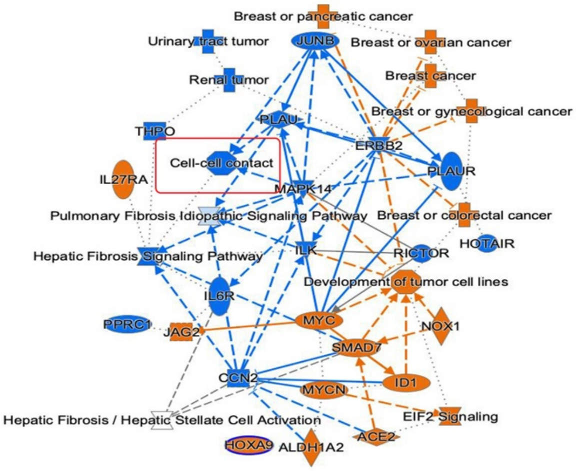

D

DN-Wnt1 E7

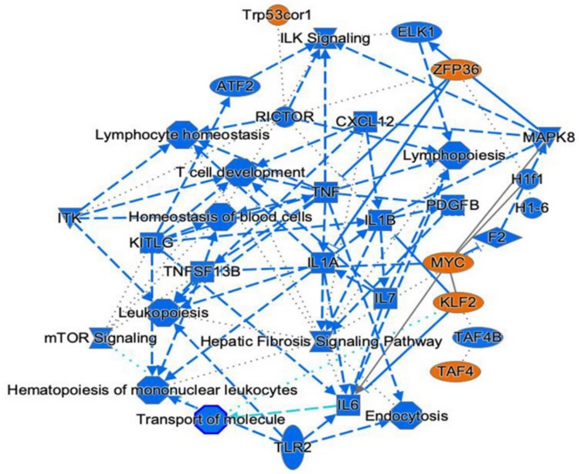

E

DN-Wnt1 E9

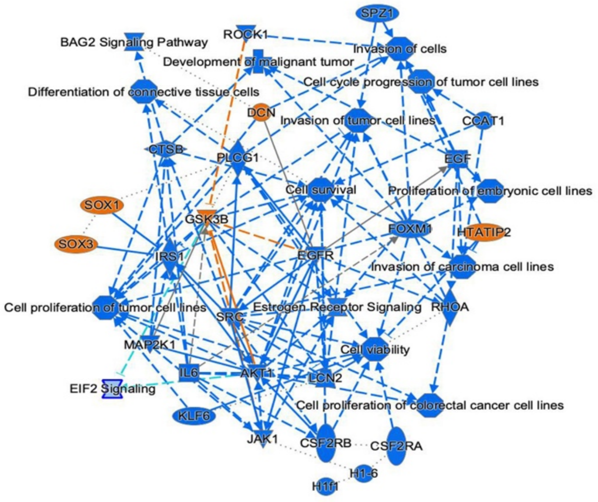

Supp. Figure 9. Ingenuity pathway analysis (IPA) of epithelial cell clusters from tumors with reduced IGF1R compared to Wnt1. A. Table of highest fold changes of EMT genes from DN-Wnt1 compared to Wnt1 tumors using an RT2 EMT profile assay. **B-E.** Graphical summaries from IPA in Cluster E0 **(B)**, E2 **(C)**, E7 **(D)** and E9 **(E)** from DN-Wnt1 compared to Wnt1 tumors. Blue=downregulated; orange=upregulated.

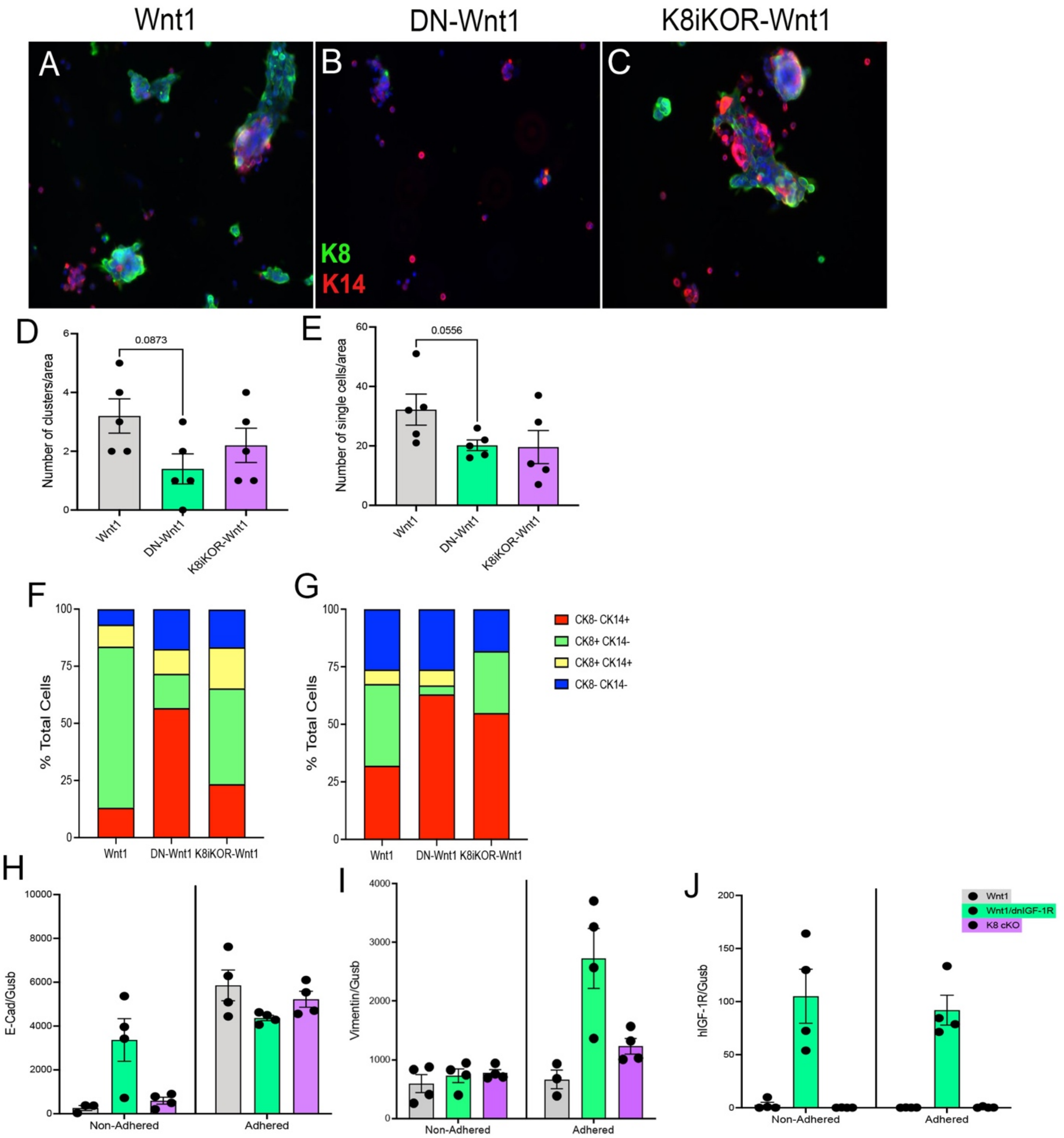

Supp. Figure 10. Adhesion is altered in tumors with attenuated IGF1R *in vitro*. A-C. Representative images of immunofluorescence for keratin 8 (K8) and keratin 14 (K14) from primary Wnt1 **(A)**, DN-Wnt1 **(B)**, or K8iKOR-Wnt1 **(C)** tumor cells attached to collagen coated plates after 10 hours of incubation. K8=green; K14=red **D-E.** Quantification of clusters **(D)** or single cells **(E)** from primary tumor cells attached to collagen coated plates after 10 hours. *Statistic:* Mann-Whitney U test **F-G.** Quantification of cell types making up the clusters **(F)** or single cells **(G)** from primary tumor cells attached to collagen matrix after 10 hours of incubation. Red=K14^{+} cells, Green=K8^{+} cells, Yellow=K8^{+}/K14^{+} cells, Blue=Keratin negative cells **H-J.** qRT-PCR for E-cadherin **(H)**, Vimentin **(I)**, and the *dnIGF1R* transgene **(J)** in primary tumor epithelial cells non-adherent and adherent to collagen after 10 hours.

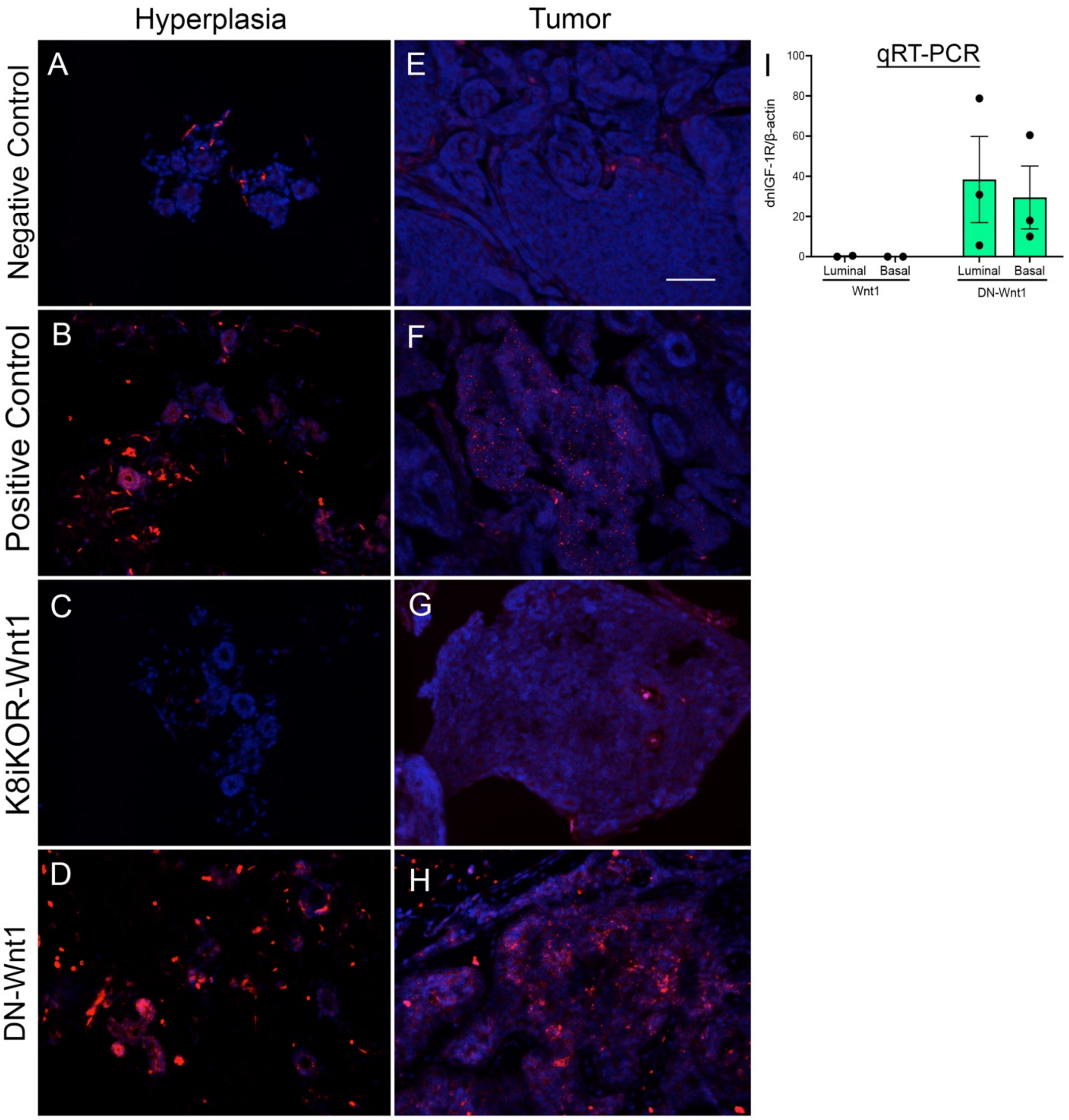

Supp. Figure 11. The *dnIGF1R* transgene is expressed in luminal and basal epithelial cells in hyperplastic mammary gland and tumor tissue. A-D. RNAscope representative images in hyperplastic mammary glands for a negative probe control **(A)**, positive housekeeping gene probe control **(B)**, and human *Igf1r* probe (dominant-negative *Igf1r* transgene) in negative K8iKOR-Wnt1 **(C)** and positive DN-Wnt1 **(D)** tissue. **E-H.** RNAscope representative images in mammary tumors as for negative probe control **(E)**, positive housekeeping gene probe control **(F)**, and human *Igf1r* probe (dominant-negative *Igf1r* transgene) in negative K8iKOR-Wnt1 **(G)** and positive DN-Wnt1 **(H)** tissue. Sections are representative of 4 different hyperplasias or tumors per group, scale bar=50 µm. **I.** RT-PCR for the human IGF-1R transgene in sorted primary tumor cells from Wnt1 and DN-Wnt1 tumors.

Gene Target	Forward Primer 5’ to 3’	Reverse Primer 5’ to 3’
Mouse CyclinD1	QuantiTect Qiagen Catalog No. 249900	
Mouse E-cadherin	GCTCTCATCATCGCCACAG	GATGGGAGCGTTGTCATTG
Mouse GAPDH	GATGCCCCCATGTTTGTGAT	GGTCATGAGCCCTTCCACAAT
Mouse Gusb	CAACGCCAAATATGATGCAG	TGCGTCTTATACCAGTTCTCAAAC
Mouse IGF-1R	CACAGCTGCAACCACGAG	GGGATATCATCTGCTCCTTCTG
Mouse IGF-1R Exon 11	CGCCTGGAAAACTGCACG	GCAGGGGATACAGTACATGTTT
Mouse K14	GCATCTACTTGCTGGACATCAG	AGGATACCCCAAGCCACTG
Human β-Actin	AGCCATGTACGTTGCTATCCA	ACCGGAGTCCATCACGATG
Human IGF-1R	GGCACAATTACTGCTCCAAAGAC	CAAGGCCCTTTCTCCCCAC

Table 1: List of qRT-PCR primers.
