# Supplementary figures and images for: Breast tumor IGF1R regulates cell adhesion and metastasis: alignment of mouse single cell and human breast cancer transcriptomics
Source: Front Oncol. 2022 Dec 7;12:990398. doi: 10.3389/fonc.2022.990398 (PMC9769962; doi:10.3389/fonc.2022.990398)

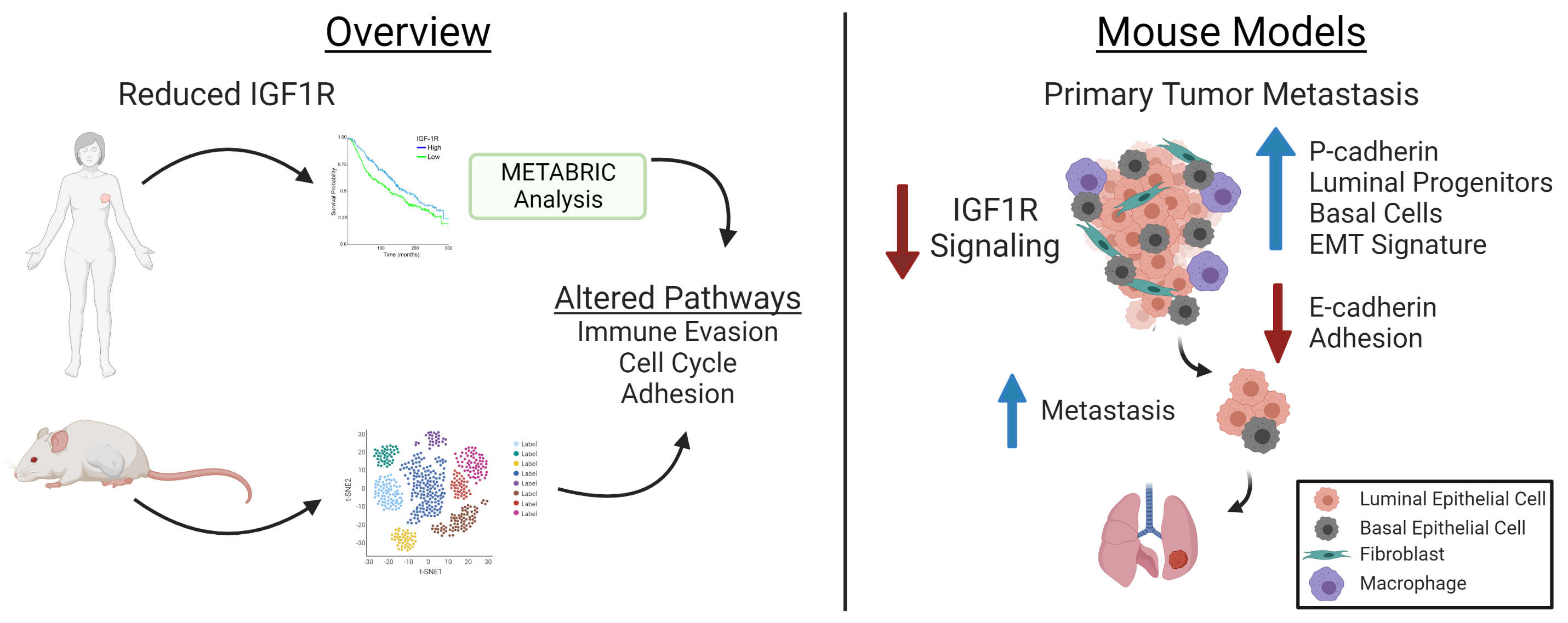

Supplement: Supplementary file 2 [file Image_1.tif]
